# Interpretable and granular video-based quantification of motor characteristics from the finger-tapping test in Parkinson’s disease

**DOI:** 10.1038/s41531-026-01307-w

**Published:** 2026-03-08

**Authors:** Tahereh Zarrat Ehsan, Michael Tangermann, Yağmur Güçlütürk, SooYoon Shin, King Chung Ho, Bastiaan R. Bloem, Luc J. W. Evers

**Affiliations:** 1 Department of Neurology, Center of Expertise for Parkinson and Movement Disorders, Donders Institute for Brain, Cognition and Behaviour, Radboud University Medical Center, Nijmegen, The Netherlands; 2 Department of Artificial Intelligence, Donders Institute for Brain, Cognition and Behaviour, Radboud University, Nijmegen, The Netherlands; 3 Verily Life Sciences, Dallas, TX, USA

**Keywords:** Computational biology and bioinformatics, Diseases, Engineering, Health care, Mathematics and computing, Medical research, Neurology, Neuroscience

## Abstract

Accurately quantifying motor characteristics in Parkinson’s disease is crucial for monitoring disease progression and optimizing treatment strategies. The finger-tapping test is a standard motor assessment. Clinicians visually evaluate a patient’s tapping performance and assign an overall severity score based on tapping amplitude, speed, and irregularity. Simultaneous video recording during the standard test enables a more objective, continuous quantification of detailed motor characteristics, thereby reducing the subjectivity and inter-rater variability inherent in clinical evaluations. This paper introduces a computer vision-based method for quantifying granular PD motor characteristics from video recordings. Four sets of clinically relevant features are proposed to characterize *hypokinesia*, *bradykinesia*, *sequence effect*, and *hesitation-halts*. We evaluate our approach on video recordings and clinical evaluations of 446 people with PD from the Personalized Parkinson Project. Using principal component analysis with varimax rotation, we show that the extracted features largely align with the four clinically defined motor deficits, while additionally revealing finer-grained substructures within the *sequence effect* and *hesitation-halts* domains. In addition, we have used these features to train machine learning classifiers to estimate the Movement Disorder Society Unified Parkinson’s Disease Rating Scale (MDS-UPDRS) finger-tapping severity score. Compared to state-of-the-art approaches, our method achieves a higher accuracy in MDS-UPDRS score prediction, while still providing an interpretable quantification of individual finger-tapping motor characteristics. In addition, we present the first large-scale dataset of finger-tapping, comprising 4073 video recordings. In summary, the proposed framework provides a practical solution for the objective assessment of PD motor characteristics, that can potentially be applied in both clinical and remote settings. Future work is needed to assess its responsiveness to symptomatic treatment and disease progression.

## Introduction

Parkinson’s disease (PD) is the second most common neurodegenerative disorder worldwide and is characterized by a progressive loss of dopamine-producing neurons in the brain^1^. Among its hallmark motor signs, bradykinesia is the most prevalent, affecting approximately 80 % of individuals with PD^2^. Traditionally, bradykinesia has referred to the slow execution of movement, reduced amplitude, and a progressive decrement in speed and amplitude during repetitive actions. However, recent studies^3,4^ suggest that bradykinesia includes potentially distinct motor characteristics with discrete pathophysiological mechanisms. To reflect this, a new revised definition of bradykinesia^3^ has been proposed which identifies four key motor characteristics that define the motor phenotype of PD and atypical parkinsonism: hypokinesia, bradykinesia, sequence effect, and hesitation-halts. In this framework, bradykinesia is defined as the slowness of movement execution, while hypokinesia refers to a reduction in movement amplitude. The sequence effect describes a progressive reduction in amplitude and speed during repetitive tasks. Hesitation-halts reflect irregularities in movement rhythm, including pauses, hesitations, and fluctuations in timing and amplitude. Bologna et al.^3^ emphasized that these four mentioned PD deficits do not share a common pathophysiological background as they respond differently to dopaminergic drugs or deep brain stimulation (DBS). Also, Giulia et al.^5^ argued that specific combinations of these deficits may aid differential diagnosis. For example, the presence of bradykinesia alongside a sequence effect is highly indicative of Parkinsonism, whereas isolated bradykinesia is a non-specific finding that may occur in a range of neurological conditions^5^. Notably, the sequence effect typically is absent in disorders such as essential tremor^6,7^ and progressive supranuclear palsy (PSP)^8^. As a result, recent literature^9^ recommends that these motor deficits be reported separately to improve the precision of disease monitoring and facilitate the development of targeted treatment strategies.

The finger-tapping test is a widely used clinical tool for assessing PD motor characteristics^10^. During the test, patients are instructed to tap their thumb and index finger as widely and rapidly as possible for a period of 10 seconds, while clinicians visually evaluate their performance based on specific criteria outlined in the Movement Disorder Society Unified Parkinson’s Disease Rating Scale (MDS-UPDRS, see Table 1). The finger-tapping test results in a single score that reflects the severity of motor impairment. However, clinical ratings might be subjective and prone to inter-rater variability, as different clinicians may interpret subtle movement abnormalities differently^11,12^. Moreover, the final score summarizes the overall performance and does not distinguish between different motor characteristics that may be present during the task. In practice, it can be challenging to reliably identify and differentiate all relevant motor deficits through visual inspection alone^13,14^. These limitations highlight the need for an objective and automated assessment method capable of providing a standardized and quantitative evaluation of individual PD motor characteristics. Such a system could improve clinical reliability and offer a deeper insight into specific movement abnormalities.

**Table 1:** Movement Disorder Society Unified Parkinson’s Disease Rating Scale (MDS-UPDRS) scoring criteria for the finger-tapping test

Score	Scoring Criteria
0	Without any problem
1	Any of the following:
	a) The regular rhythm is broken with one or two interruptions or hesitations of the tapping movement;
	b) Slight slowing;
	c) The amplitude decrements near the end of the 10 taps
2	Any of the following:
	a) 3 to 5 interruptions during tapping;
	b) Mild slowing;
	c) The amplitude decrements midway in the 10-tap sequence
3	Any of the following:
	a) More than 5 interruptions or at least one longer freeze in ongoing movement;
	b) Moderate slowing;
	c) The amplitude decrements starting after the 1st tap
4	Cannot or can only barely perform the task because of slowing, interruptions, or decrements

In this test, patients are instructed to tap the index finger on the thumb 10 times, as fast and as large as possible.

Recent advances in computer vision have led to new approaches for automating PD assessment^15^. Video-based methods leveraging computer vision can offer a contactless, non-invasive and highly accessible solution^16^. These methods could realize low-cost assessments using widely available devices, such as smartphones or laptop cameras, and allow for a standardized and frequent evaluation of the finger-tapping test^17^. However, existing approaches typically focus on reproducing imperfect clinical evaluations using black-box machine learning models. Thus, they lack clinical interpretability and do not provide insights into the individual motor characteristics ^18^. Moreover, most of these approaches have been developed using small video datasets only^19,20^, which had been recorded under highly controlled conditions^21,22^. As a result, it remains unclear how well the reported assessment performances will transfer to real-world clinical- or even home environments.

In recent years, several studies have explored methods to quantify PD severity using finger-tapping tests^23,24^. They can be categorized into two groups: (1) methods focusing on specific motor characteristics and (2) methods focusing on MDS-UPDRS score classification. Approaches in the first category have sought to isolate distinct PD characteristics. For example, Zhao et al.^25^ applied clustering to time-series signals to detect amplitude decrements in finger-tapping tasks. Similarly, Heye et al.^26^ focused on extracting tapping frequency, amplitude, and rhythm variability to quantify bradykinesia and hesitation-halts. In contrast, the second group aimed to classify MDS-UPDRS scores without explicitly categorizing features into distinct groups of motor deficits. For instance, Islam et al.^27^ used the Mediapipe^28^ model to identify three key points (wrist, thumb tip, and index finger positions) from each video frame. Two lines are drawn from wrist to thumb and wrist to index finger and the angle between these two lines is computed. From this angular displacement signal, they derived features and trained a light gradient boosting machine (LightGBM) to classify MDS-UPDRS scores. Although it achieves high accuracy, the high feature dimensionality (116 features) without clear grouping for distinct motor characteristics challenges the clinical interpretability. Similarly, Guarin et al.^29^ leveraged angular displacements as features, and applied logistic regression to classify videos. Guo et al.^30^ used depth video to capture 3D hand movement representations. Hand regions were identified using the you only look once (YOLO) object detection model^31^. Thumb and index finger key points were used to extract features such as amplitude, velocity, and frequency. A support vector machine (SVM) was trained on these features to classify MDS-UPDRS scores. Although depth cameras provide more precise representation, the higher cost and limited availability of depth cameras make this approach impractical for home-based monitoring. Deng et al.^20^ expanded previous methods by analyzing all fingers (including middle, little, and ring fingers) and calculated first- and second-order derivatives to obtain velocity and acceleration metrics for each finger. However, their classification was limited to only two MDS-UPDRS severity levels. More recently, Kim et al. introduced the TULIP dataset^32^, the first publicly available multi-camera dataset for PD motor assessment. By using synchronized recordings from six cameras, TULIP enables accurate 3D pose reconstruction and extraction of kinematic features, resulting in improved MDS-UPDRS score prediction for finger tapping and gait compared with 2D feature-based models. However, the reliance on a multi-camera laboratory setup limits its direct applicability to home-based monitoring. In another recent study, Amprimo et al.^33^ employed a semi-supervised self-training framework that leveraged unlabelled finger-tapping videos. Their method improved cross-dataset performance, underscoring the value of unlabelled data in mitigating domain shifts in real-world settings.

Several studies^18,34,35^ have explored deep learning models to estimate MDS-UPDRS scores. Williams et al.^19^ used convolutional neural networks (CNNs) combined with optical flow to extract motion features and classify MDS-UPDRS scores. Yang et al.^36^ trained a small deep neural network (DNN) using tapping rate, frozen time, and amplitude variation to predict four MDS-UPDRS severity levels. Raw hand key points were fed into a multi-channel 1D CNN to capture temporal patterns at different scales and predict MDS-UPDRS scores^35^. More advanced CNN-based models have been proposed, such as the three-stream architecture by Lu et al.^34^, which processes joint distance, slow-motion, and fast-motion features for severity classification. A related study by Lu et al.^18^ designed a three-stream network incorporating pose, motion, and geometric features, and leveraged a 1D CNN for MDS-UPDRS score classification. These approaches have demonstrated high accuracy. However, as they primarily aim to replicate MDS-UPDRS scores, they remain constrained by the subjectivity of the score. Instead, the broader aim is to leverage objective, video-based features to achieve a more granular and interpretable understanding of individual motor characteristics in PD. This could enable earlier detection of subtle impairments, better monitoring of symptom progression, and a more personalized assessment of treatment effects. While correlating with existing clinical scores remains essential to confirm their validity, the true potential of video-based assessment lies in surpassing the limitations of subjective ratings by offering consistent, quantitative, and clinically meaningful insights.

**Fig. 1:**
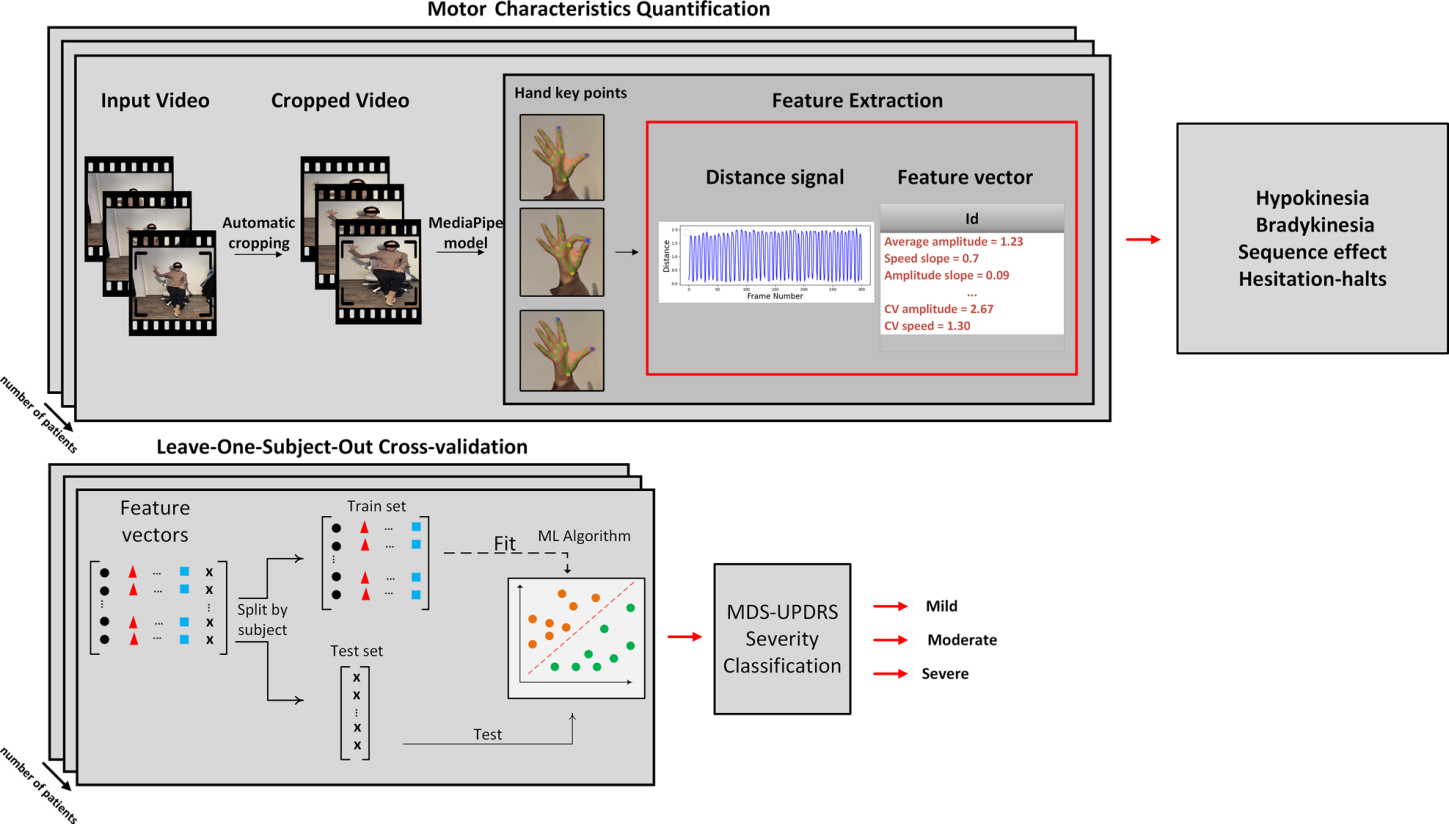
Block diagram describing the analysis pipeline of the proposed method. The system detects key points---including the index finger, thumb, and wrist---in each video frame and tracks them throughout the video sequence. Next, a time-series signal is generated, representing the distance between the thumb and index finger, which reflects the patient’s ability to fully open their fingers. This signal is scaled by the palm length to account for variations in the patient’s distance and angle to the camera. Features are extracted from the signal to assess PD severity across four main domains: hypokinesia, bradykinesia, sequence effect, and hesitation-halts. Per video, the Movement Disorder Society Unified Parkinson’s Disease Rating Scale (MDS-UPDRS) severity score values are then estimated by machine learning classifiers, which had been trained on these features. The classification performances are estimated using a leave-one-subject-out cross-validation scheme.

To address these challenges, this paper presents a clinically interpretable video-based framework for quantifying distinct PD motor deficits from finger-tapping recordings. Unlike prior approaches that primarily focus on reproducing MDS-UPDRS scores using high-dimensional or black-box models, our framework is explicitly structured around the recently revised clinical definition of bradykinesia^3^. We design a low-dimensional and clinically interpretable feature set that separately quantifies hypokinesia, bradykinesia, sequence effect, and hesitation-halts, enabling granular characterization of motor impairment. Importantly, we validate this framework in a large-scale clinical dataset comprising 4,073 videos from 446 individuals with PD, and further assess cross-dataset generalizability using an independent external cohort. The block diagram in Fig. 1 provides an overview of the proposed processing pipeline. First, the input video is pre-processed to extract hand key points using Mediapipe^28^. Using these, a time-series signal representing the distance between the thumb tip and the index finger tip is generated. The resulting distance values are used to extract clinically interpretable features, which allow for quantification of individual motor characteristics, based on a recently updated clinical definition of bradykinesia^3^. We apply principal component analysis (PCA) with varimax rotation to assess whether these video-based features co-vary according to the four proposed clinical motor characteristics, and to reveal potential additional components. To assess clinical validity, We evaluate whether these features can be used to train machine learning classifiers to estimate the MDS-UPDRS finger-tapping severity score.

## Results

To demonstrate the clinical validity of our approach, we analyzed the extracted features from multiple perspectives. First, we assessed whether the features align with expert-rated MDS-UPDRS scores to confirm that they capture clinically relevant patterns. Next, we used PCA with varimax rotation to explore the underlying structure of motor characteristics and to validate or refine clinical groupings. We then evaluated how well the features support automated classification of MDS-UPDRS scores using multiple machine learning models. Finally, we benchmarked our method against state-of-the-art approaches.

### Alignment of video-based features with clinical finger-tapping assessments

**Fig. 2:**
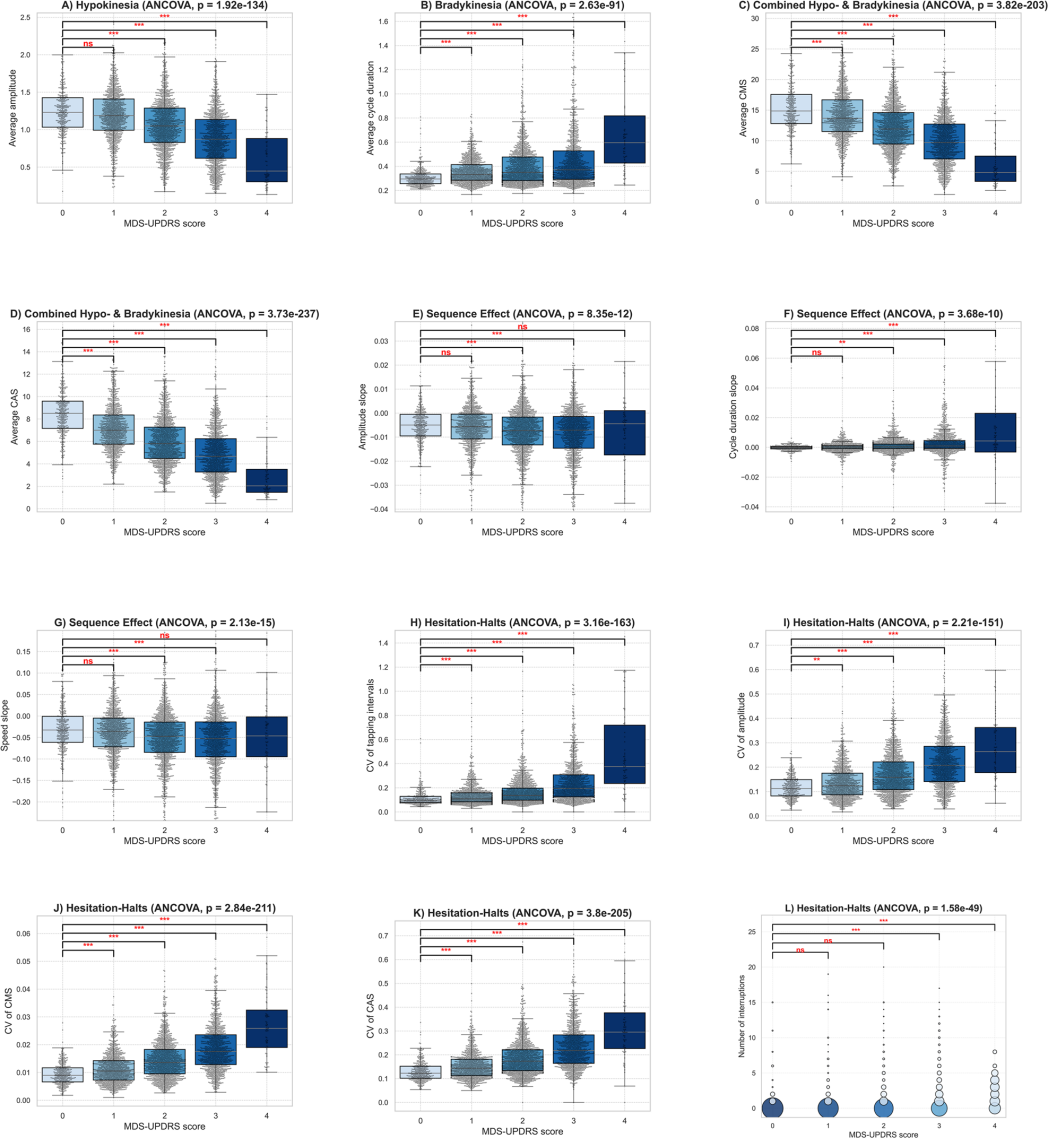
Distribution of clinically interpretable features across Movement Disorder Society Unified Parkinson’s Disease Rating Scale (MDS-UPDRS) finger-tapping scores. For our PPP dataset, each subplot shows the distribution of a specific feature across MDS-UPDRS scores (0-4), highlighting its relation to motor impairment severity. **A** Hypokinesia: average amplitude, measuring hand opening ability. **B** Bradykinesia: Average cycle duration. **C**,**D** Combined hypo- and bradykinesia: Average CMS and CAS. **E**–**G** Sequence effect: amplitude slope, cycle duration slope, speed slope. **H**–**l** Hesitation-halts: coefficient of variation (CV) in cycle duration, amplitude, Cycle Maximum Speed (CMS), Cycle Average Speed (CAS), and number of interruptions. Each feature reflects a clinically relevant aspect of motor impairment, with distributions aligning with increasing disease severity. Significance levels are indicated as: ^*^p < 0.05, ^**^p < 0.01, ^***^p < 0.001 (Bonferroni-corrected). Subplots (**A**–**K**) use boxplots: the horizontal line within each box indicates the median; the lower and upper box boundaries correspond to the first (Q1) and third (Q3) quartiles; and the whiskers span 1.5 times the interquartile range. Subplot (**L**) uses a bubble plot to represent the interruption feature: each bubble corresponds to a specific number of interruptions, while bubble size and color jointly encode the proportion of participants within each UPDRS group exhibiting that value. Statistical tests are adjusted for age and gender.

To characterize the relationship between the extracted video-based features and clinical scores, we first assessed how well they align with clinical MDS-UPDRS finger-tapping scores. This analysis determines whether the proposed features meaningfully capture the motor impairments typically observed during clinical assessments. We quantified 12 features measuring four groups of finger-tapping motor characteristics: hypokinesia, bradykinesia, sequence effect and hesitation-halts. Also, two other features capture the combined effect of hypokinesia and bradykinesia. The distribution of these features across MDS-UPDRS scores of the finger-tapping task (0 to 4) is presented in Fig. 2. For statistical evaluation, a one-way analysis of covariance (ANCOVA) was applied to each feature to test for group differences across the five MDS-UPDRS scores, with age and sex included as covariates. When the ANCOVA indicated significant variability, pairwise post-hoc comparisons were conducted between each group with scores 1-4 and the group with score 0. Bonferroni correction was applied to adjust for multiple comparisons. Statistical significance levels with corrected thresholds are indicated in the figures. Subplot (a) focuses on the hypokinesia feature “average amplitude", which measures the extent to which patients can open their fingers during the task. This feature decreased significantly with increasing MDS-UPDRS scores (*p* < 0.001). Subplot (b) presents the bradykinesia feature. The average cycle duration captures the time required to complete each cycle of tapping, which is significantly higher for patients with a MDS-UPDRS score of 4 (*p* < 0.001). Average cycle max speed (CMS) and cycle average speed (CAS) in subplots (c) and (d) capture both hypokinesia and bradykinesia, which progressively decrease as MDS-UPDRS scores increase (*p* < 0.001). Subplots (e), (f), and (g) present the sequence effect features, which capture changes in amplitude, cycle duration, and speed within a task session. For amplitude and speed slope, the overall ANCOVA revealed a significant group effect; however, post-hoc pairwise comparisons did not show significant differences between all pairs. Cycle duration slope exhibited a trend toward increasing cycle duration with higher disease severity. Subplots (h) to (k) show the hesitation-halts features. The coefficients of variation (CV) capture irregularity in amplitude, cycle duration and speed over time. Higher MDS-UPDRS scores exhibit a larger variability in amplitude, cycle duration and speed (*p* < 0.001). For the final hesitation-halts feature, number of interruptions, we replaced the boxplot with a bubble plot to more appropriately visualize this discrete metric. The subplot shows that the majority of participants with scores 0 to 2 exhibit zero interruptions, whereas the probability of experiencing interruptions significantly increases with scores 3 and 4 (*p* < 0.001). Together, these results show that all video-based features, except for the sequence effect features, show the expected relationship with the clinical evaluation of the finger-tapping task.

**Fig. 3:**
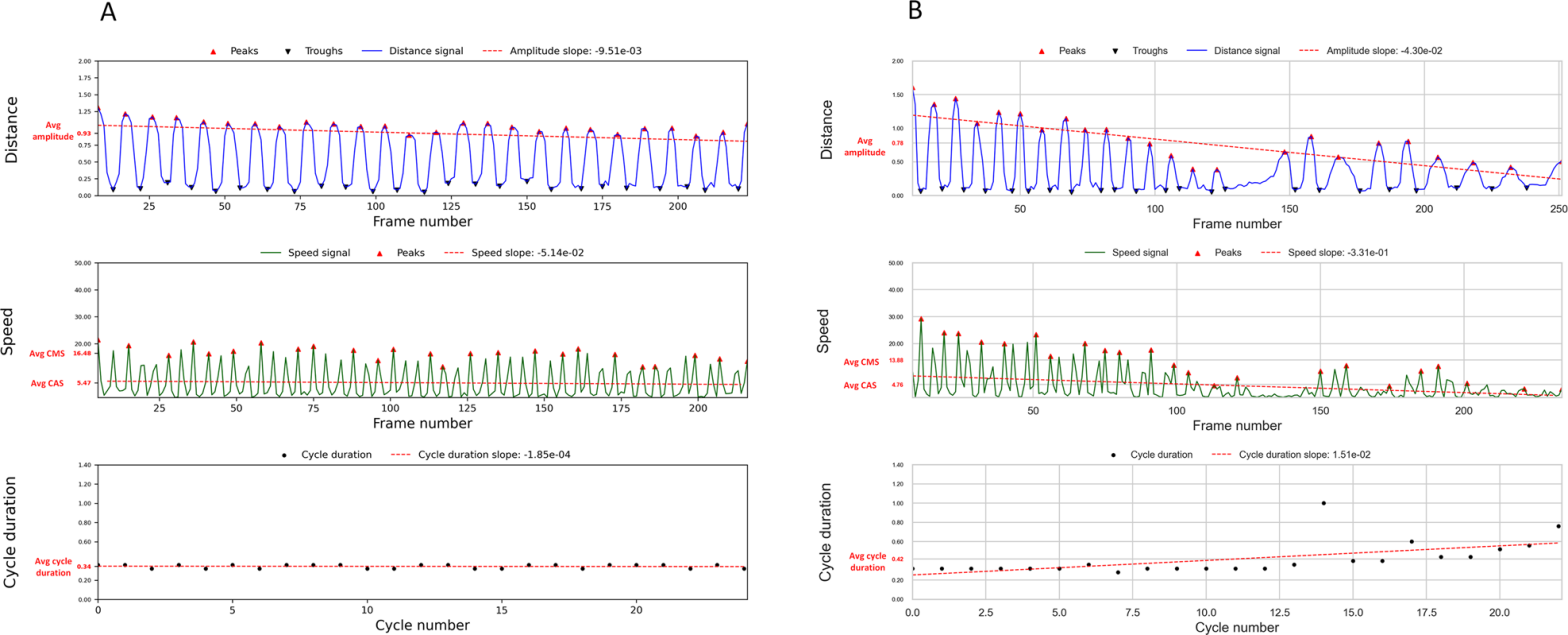
Amplitude, speed, and cycle duration for two patients with Movement Disorder Society Unified Parkinson’s Disease Rating Scale (MDS-UPDRS) scores of 0 and 3. **A** For the patient with an MDS-UPDRS score of 0, the distance signal demonstrates high-amplitude tapping. The speed signal shows stable, high tapping speed, reflecting the ability to maintain steady tapping throughout the task. The average cycle duration for this patient is 0.34 seconds. **B** The patient with an MDS-UPDRS score of 3 exhibits lower tapping amplitudes and markedly reduced tapping speed, which decreases over time. The cycle duration is prolonged, with an average duration of 0.42 seconds.

Figure 3 show the distance and speed signals and features derived thereof for two illustrative examples: one patient with a MDS-UPDRS finger-tapping score of 0 (minimal motor impairment), and one patient with a MDS-UPDRS score of 3 (severe motor impairment). The plots show how amplitude, speed, and cycle duration differ between these two cases.

### Redefining motor characteristics categories based on PCA-varimax

**Fig. 4:**
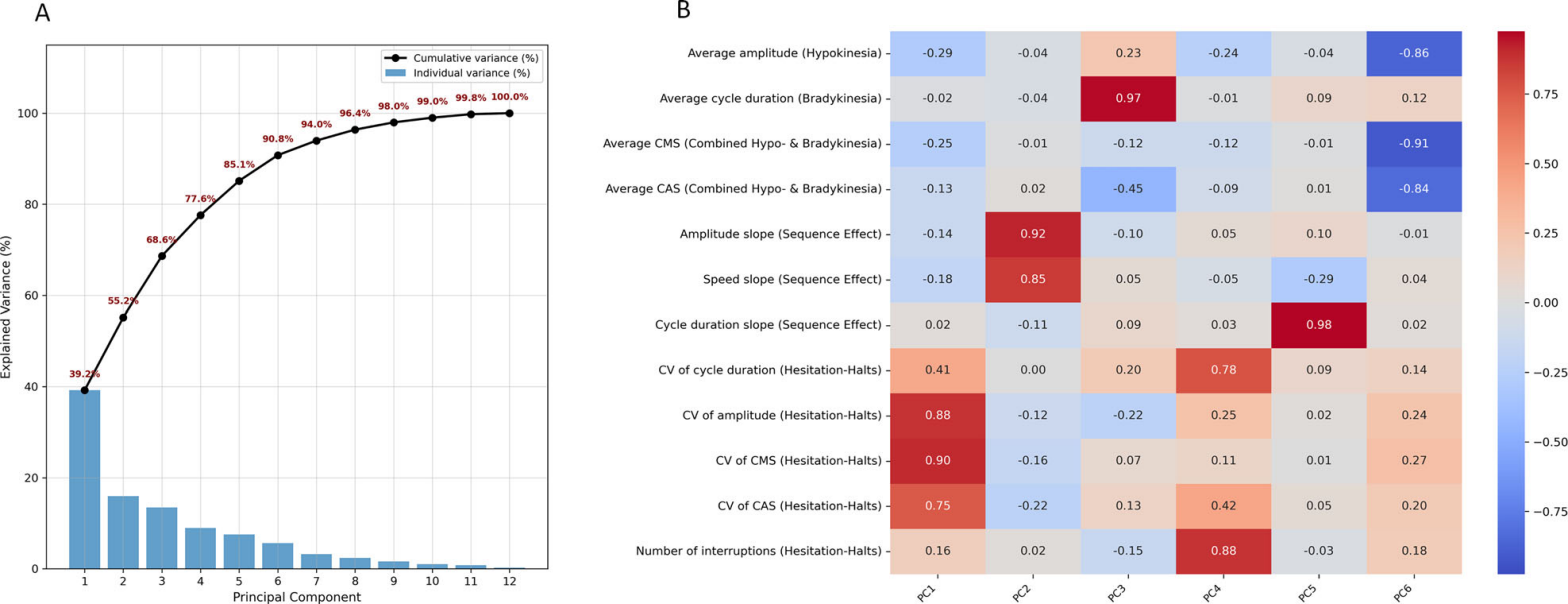
Principal component analysis of finger-tapping features. **A** Showing the proportion of variance explained by each principal component (blue bars) and cumulative variance (black line) on our PPP dataset. Based on the elbow criterion, six components were selected, together accounting for approximately 90.8 % of the total variance. **B** Varimax-rotated PCA loadings illustrating the underlying feature structure.

To examine whether the motor deficit categories proposed by Bologna et al.^3^ are supported by the data, we applied principal component analysis (PCA) with varimax rotation^37^ to the complete set of extracted features. This analysis aimed to (i) determine the dimensionality required to explain the variability in the data and (ii) assess whether the four hypothesized clinical domains—hypokinesia, bradykinesia, sequence effect, and hesitation-halts—emerge as distinct components. Figure 4 shows the scree plot and the proportion of variance explained by each principal component. Based on the elbow criterion, six components were selected and subsequently rotated to enhance interpretability. Overall, the extracted components partially align with the four clinically defined domains. Notably, the results indicate that sequence effect and hesitation-halts may not be unitary constructs. Specifically, the sequence effect domain separates into two distinct components: one capturing amplitude and speed slope, and another reflecting cycle duration slope. Likewise, the hesitation-halts domain decomposes into two components with a similar internal structure. One component is dominated by variability in amplitude and speed-related measures—specifically the coefficients of variation of amplitude, CAS, and CMS—while the other is characterized by variability in cycle duration and the number of interruptions. The stability of this component structure was further confirmed using bootstrap resampling, which demonstrated high consistency across resampled datasets (Supplementary Fig. 2). Overall, these findings suggest that, while the four clinically defined domains remain informative, a six-dimensional representation may capture additional structure in finger-tapping motor impairments.

### Finger-tapping MDS-UPDRS severity classification

**Fig. 5:**
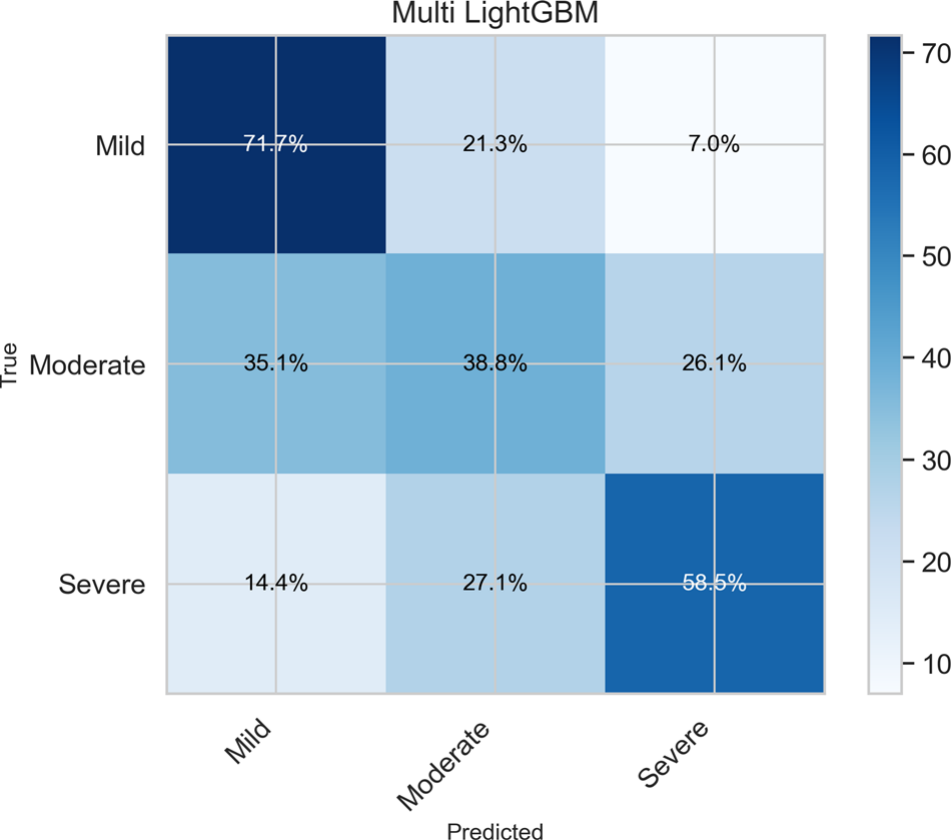
Confusion matrix for the multi-class LightGBM classifier showing the distribution of predicted versus true classes for the PPP dataset. Percentages indicate predicted versus true severity classes. Correct predictions are shown along the diagonal, while most errors occur between adjacent severity levels.

We evaluated whether the video-based features can be used to train machine learning classifiers to estimate the MDS-UPDRS finger-tapping score. Classification performance was assessed using accuracy, balanced accuracy, macro precision, and macro F1-score, as summarized in Table 2. Differences between classifiers (random forest, logistic regression, and LightGBM) and between multi-class and ordinal classification strategies were generally small (< 1 %). Nevertheless, the multi-class LightGBM model achieved the highest performance across multiple metrics, with an accuracy of 56.44 % and balanced accuracy of 56.33 %. Based on this overall trend, the multi-class LightGBM classifier was selected for subsequent analyses. The confusion matrix for the multi-class LightGBM model (Fig. 5) shows that the majority of misclassifications occur between neighboring severity classes. Confusion matrices for the other models are provided in the Supplementary Fig. 6. Separate evaluations for the off- and on-medication conditions are reported in the supplementary Table 1.

**Table 2:** Model performance comparison

A. Evaluation on Personalized Parkinson Project (PPP) dataset								
	Accuracy		Balanced Acc.		Macro Precision		Macro F1	
Model	Multi	Ordinal	Multi	Ordinal	Multi	Ordinal	Multi	Ordinal
Logistic regression	56.39	56.10	56.19	55.84	56.17	**56.52**	55.75	55.75
LightGBM	**56.44**	56.12	**56.33**	56.05	55.83	55.70	**55.83**	55.69
Random forest	55.17	55.22	55.13	55.19	54.79	54.39	54.84	54.55
**Baselines**								
Random guess	33.32		33.32		33.32		33.30	
Majority class	34.84		33.33		11.61		17.23	

B. Evaluation on an independent external TULIP dataset								
	Accuracy		Balanced Acc.		Precision		F1-score	
Model	Multi	Ordinal	Multi	Ordinal	Multi	Ordinal	Multi	Ordinal
Logistic regression	54.55	**59.09**	55.15	61.82	55.86	58.66	49.09	56.86
LightGBM	**59.09**	**59.09**	61.82	65.45	61.11	**72.62**	56.64	**60.00**
Random forest	54.55	**59.09**	**66.06**	61.82	66.67	69.84	52.99	57.50
**Baselines**								
Random guess	33.38		33.26		33.10		31.46	
Majority class	50.00		33.32		16.67		22.22	

Bold values indicate the overall highest value for each evaluation metric, considering both multi and ordinal settings.

We further evaluated cross-dataset generalizability by training models on PPP and testing them on video recordings from the TULIP dataset^32^. Performance was comparable to, and in some cases higher than, the within-PPP evaluation. The best-performing model (multi-class LightGBM) achieved a balanced accuracy of 61.82 %, a macro precision of 61.11 %, and a macro F1-score of 56.64 %. These results indicate that models trained on PPP generalize well to the TULIP dataset, despite differences in data recording setups.

### Comparison with related work for MDS-UPDRS finger-tapping severity classification

**Fig. 6:**
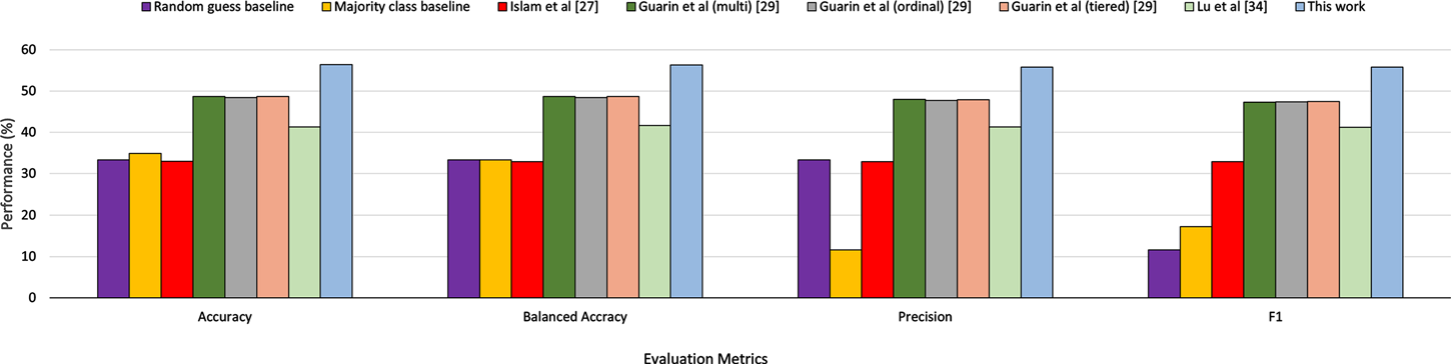
Comparison of the proposed method with existing state-of-the-art approaches for Movement Disorder Society Unified Parkinson’s Disease Rating Scale (MDS-UPDRS) finger-tapping score classification. Results for the method by Islam et al.^27^ are obtained by using the official implementation from the authors' GitHub repository. To ensure fair comparison, their hyperparameters are re-tuned on our dataset using the optuna library. For Guarin et al.^29^ and Lu et al.^34^, official codes are not publicly available; therefore, these methods are re-implemented based on their publications. All methods are trained and evaluated on our dataset under identical experimental settings using leave-one-subject-out cross-validation.

Figure 6 presents a detailed comparison of our MDS-UPDRS classification method with state-of-the-art approaches. Our proposed method consistently outperforms related work across all evaluated metrics. Our method achieves an accuracy of 56.44 %, a significant improvement of 15.87 % over the highest accuracy of 48.71 % of alternative models^29^ (*p* = 1.07 × 10^−9^). For balanced accuracy, our method reaches 56.33 %, which represents an improvement of 15.69 % compared to the best-performing alternative model (balanced accuracy of 48.69 %)^29^ (*p* = 1.39 × 10^−8^). Also, macro precision of 55.83 % (*p* = 1.04 × 10^−6^) and macro F1-score of 55.83 % (*p* = 3.44 × 10^−9^), demonstrated statistically significant improvement. All reported *p*-values were adjusted for multiple comparisons using Bonferroni correction.

To better understand the factors underlying the superior performance of our approach, we investigated the impact of differences in signal representation across pipelines. A key distinction from prior work is our use of distance-based signals, which are inherently less sensitive to variations in camera viewpoint compared with angle-based representations^27,29^. To quantify the effect of this design choice, we conducted an analysis in which identical feature sets were extracted separately from distance-based and angle-based signals, while keeping the classifiers and evaluation protocol fixed. The corresponding results, reported in Table 3, show that features derived from distance-based signals consistently outperform those based on angle representations across all classifiers and evaluation metrics. The difference between distance- and angle-based signal extraction is illustrated in Supplementary Fig. 3 and Fig. 4.

**Table 3:** Comparison of classification metrics using distance-based and angle-based features on the PPP dataset

Features	Classifier	Accuracy (%)	Balanced Accuracy (%)	Macro Precision (%)	Macro F1 (%)
Distance-Based	Logistic regression	**56.39**	**56.19**	**56.17**	**55.75**
Angle-Based	Logistic regression	50.18	50.02	49.33	48.03
Distance-Based	LightGBM	**56.44**	**56.33**	**55.83**	**55.83**
Angle-Based	LightGBM	50.03	50.11	48.71	48.97
Distance-Based	Random forest	**55.17**	**55.13**	**54.79**	**54.84**
Angle-Based	Random forest	49.00	49.03	48.12	48.37

Three classifiers (logistic regression, LightGBM, and random forest) are trained on two different input modalities: distance signal and angle signal. The same feature extraction pipeline was applied separately to the distance and angle signals.

Bold values indicate the higher performance between distance-based and angle-based features for each classifer and evaluation metrics.

### Analysis of misclassified samples

To better understand the classification errors made by our proposed model, we identified all videos for which the model’s predicted severity differed by more than one point from the clinical rating. From this set, a random subset of 34 videos was selected for further analysis. These videos were re-scored by an independent assessor who was blinded to both the clinical ratings and the model-generated predictions. Of the re-scored videos, 27 % of the new scores aligned with the model’s predictions, 31 % aligned with the original assessor’s ratings, and 42 % fell between the two.

## Discussion

In this work, we translated the recently updated clinical definition of bradykinesia^3^ into an AI-based framework for the granular quantification of PD motor deficits during the finger-tapping task. We developed a set of clinically interpretable features that quantify four key motor domains: hypokinesia, bradykinesia, sequence effect, and hesitation-halts, allowing for a structured assessment of distinct movement deficits. We evaluated their performance in a large-scale dataset of 4073 finger-tapping task recordings from 446 people with PD. The structure that we identified through principal component analysis largely corresponded with the four proposed clinical deficits, but also revealed more granular distinctions within the sequence effect and hesitation-halts deficits. We confirmed the clinical validity of our approach by showing that the proposed features can predict MDS-UPDRS bradykinesia ratings with higher accuracy than state-of-the-art methods, while still providing interpretable measures of individual motor characteristics.

We developed 12 video-based features and evaluated their relationship with MDS-UPDRS bradykinesia severity levels. Hypokinesia, bradykinesia, and hesitation-halts features significantly distinguished between these severity levels. Interestingly, whereas most features gradually worsened with increasing severity, the presence of interruptions appeared to be specific to the greater clinical severity levels. In contrast, the sequence effect features did not exhibit a consistent trend with increasing severity, and only showed small differences between different severity levels. This absent correlation with disease severity may be explained by the fact that we focused on macro-level decrements—capturing the overall trend across the entire tapping task. If a patient experiences a temporary drop in performance mid-test but later recovers, this is not reflected by our sequence effect features. Instead, such short-term fluctuations are captured by our hesitation-halts features, which reflect micro-level irregularities within the tapping sequence.

To further explore the structure of motor impairments, we applied PCA with varimax rotation. The results aligned partially with the four proposed motor deficits but revealed additional substructures. Specifically, both the sequence effect and hesitation-halts exhibited a comparable internal structure, separating into components related to amplitude and speed versus cycle duration. As a result, a six-dimensional representation was required to adequately capture the variance in the data. Future research is needed to establish the clinical relevance of these components by evaluating their ability to capture treatment effects and track disease progression.

Since clinical assessment is currently the gold standard for quantifying motor impairment in PD^14^, we evaluated whether a machine learning model trained on the video-based features could predict the MDS-UPDRS bradykinesia score. Our results outperformed existing video-based methods ^27,29^. This improvement could be attributed largely to the use of distance-based signals, which are more robust to camera viewpoint variability compared to angle-based signals commonly used in previous studies^27,29^. This suggests that distance-based signals are a more reliable and accurate approach for quantifying the finger-tapping test.

To further assess the robustness and generalizability of our approach beyond the PPP dataset used for method development, we evaluated the proposed pipeline on an independent TULIP cohort. Despite differences in recording setup and participant population, the performance remained comparable to—or slightly exceeded—our results obtained when testing on our own PPP dataset. This transfer performance on the TULIP dataset indicates that the proposed method is capable of capturing stable and generalized characteristics, rather than dataset-specific patterns. Nevertheless, given the limited sample size of the TULIP cohort, further validation in larger and more heterogeneous external datasets will be important to strengthen evidence for robustness across diverse clinical settings.

While the ability to predict MDS-UPDRS bradykinesia scores supports the clinical validity of our approach, we believe that striving for perfect prediction of currently accepted - but imperfect - clinical assessments should not be the goal. The true potential of video-based assessment lies in surpassing the limitations of subjective ratings by offering consistent, quantitative, and granular insights into distinct motor deficits.A key strength of this work lies in the unprecedented scale of its dataset— a large-scale collection of 4,073 video-recorded finger-tapping tasks from 446 people with PD. In comparison, previous studies in this field have analyzed fewer than 500 videos^19,20,27,34,38^. Moreover, focusing on interpretable feature that directly reflect key clinical motor deficits increases the utility of our approach in clinical trials and patient care. Finally, compared to wearable sensor-based approaches^39,40^, our method is entirely non-contact and does not require physical attachment of sensors to the patient’s fingers, which may simplify its use for motor assessments in clinical settings and at-home recordings.

Certain limitations must also be acknowledged. First, all videos were acquired within the same clinic and under the supervision of trained assessors. However, the recordings were not fully uniform, as data were collected across different examination rooms with varying backgrounds and room illumination, without the use of an explicit external lighting. In addition, the distance and camera angle relative to the participant were not fixed to predefined values, introducing further variability across recordings that more closely resembles real-world scenarios than a fully controlled lab environment would. Nevertheless, recordings were obtained within a clinical setting, and therefore the generalizability of the present findings to more heterogeneous and fully uncontrolled environments, such as patients’ homes, remains to be established in future work. Also, in some videos, finger key points were not visible during the entire execution of the task, which probably impacted the quality of the signal and features extracted from it. Additionally, for patients with severe impairment, the inability to perform the task properly may have led to inaccurate hand keypoint detections. For example, some patients could not raise their hands and placed them on the chair’s armrest, causing key points to be partially occluded. In addition, although MDS-UPDRS scores were assigned by trained assessors, the subjectivity of clinical ratings may introduce uncertainty in the ground truth. However, this is also the reason that developing machine learning models to predict MDS-UPDRS scores was not the main aim of our study, but rather to provide granular features to objectively quantify specific motor characteristics.

Video-based assessment of bradykinesia offers versatile applications across clinical trials (in the short term) and patient care (in the longer term). In the context of clinical trials, our system can be used to objectively monitor motor impairments in people with PD. While this might initially mainly be used as an exploratory endpoint, it may eventually be promoted to secondary or possibly even a primary endpoint if proven to be valid and representative for everyday performance. This monitoring system may also be extended to individuals in the prodromal phase of PD^41^. Detecting and tracking changes in this early stage of PD is especially important for the various ongoing clinical trials focusing on disease-modifying therapies that aim to postpone or even prevent the onset of clinically manifest PD. In these studies, tools that can sensitively capture even small changes in motor deficits will be essential for identifying treatment effects^16^. It will be interesting to see if our approach can be applied also to home-based videos, such that participants in these trials would record their test performance in their own environment. This would allow for multiple repeated assessments at home, which could significantly reduce participant burden in long-term disease-modifying trials. In terms of patient care, the proposed framework could support telemedicine by enabling remote, standardized monitoring of motor symptom severity and response to treatment^42^. This characteristic is particularly beneficial for patients with limited mobility, those living in rural or underserved areas, or individuals who face other difficulties with attending frequent in-clinic visits. By providing objective, fine-grained measurements of motor patterns, the framework could add valuable clinical information to support more accurate diagnosis and follow-up. For such a clinical implementation to take place, future research should focus on evaluating the responsiveness of extracted features to medication effects and their potential to track disease progression over time.

## Methods

### Data

Data was obtained from the Personalized Parkinson Project (PPP)^43^. In brief, PPP is a cohort study including 517 early-stage (diagnosed < 5 years ago) PD patients. Follow-up included in-clinic visits at baseline, after one year, and after 2 years, and continuous at home monitoring using a wrist-worn sensor for up to three years. In this work, we used the video-recorded MDS-UPDRS part III examinations obtained during the yearly in-clinic visits.

As part of the MDS-UPDRS part III examination, each participant completed a finger-tapping task twice: once in an “off" state and once in an “on" state. To ensure an accurate off state assessment, participants abstained from dopamine-based medications for at least 12 hours before the first recording. After completing the first recording, participants took their prescribed medication, allowing time for it to take effect, and then performed the second finger-tapping test in the on state. The video recordings were conducted using a camera with a resolution of 1280 × 720 pixels, recorded at either 25 or 50 frames per second (fps).

**Fig. 7:**
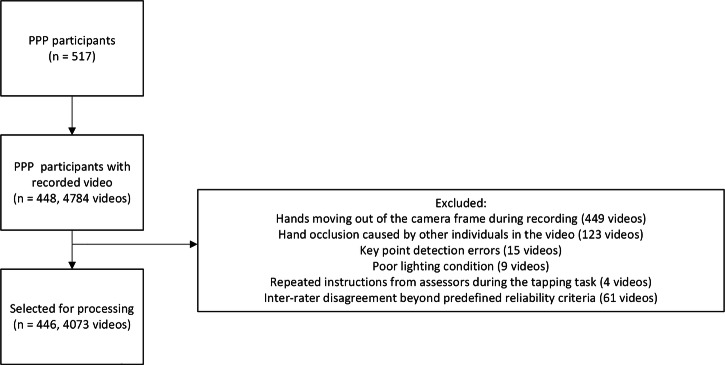
Overview of participant inclusion and video quality control for the finger-tapping dataset. The flowchart summarizes the number of available recordings, applied exclusion criteria, and the final set of videos used for analysis.

The in-person clinical evaluation of the finger-tapping tasks was performed by trained assessors following the MDS-UPDRS part III instructions. In addition, a subset of video recordings was also evaluated by trained assessors based on the video recordings. Most videos (n = 3,699) had a single clinical rating, while the remainder had between 2 and 7 ratings (58 videos with two ratings, 314 with three, 15 with four, 43 with five, 3 with six, and 2 with seven ratings). Inter-rater consistency was quantified using Krippendorff’s *α*, which yielded *α* = 0.52. Specifically, 94 % of all rater-pair disagreements were within ± 1 point, and only 6 % differed by ≥2 points (Supplementary Fig. 5). Videos exhibiting ≥2-point disagreement were excluded from the dataset to reduce the impact of potential labeling noise on model training and evaluation. The dataset flowchart is summarized in Fig. 7. After applying all quality-control criteria–including removal of videos with hand occlusion, hands moving out of frame, key point detection failures, poor lighting, repeated assessor instructions, or substantial inter–rater disagreement–a total of 4073 videos from 446 participants were retained for analysis. Of these, 2094 were recorded in the off state and 1979 in the on state.

Table 4 summarizes the demographic and clinical characteristics of the participants, including age, sex distribution, disease duration, and medication status. Additionally, it presents scores from the MDS-UPDRS parts I, III, and IV, which assess different aspects of PD progression. The distribution of MDS-UPDRS scores is also detailed in Table 5. For the classification task, the original five-point MDS-UPDRS scores of the finger tapping assessment were grouped into three clinically meaningful severity categories: *Mild* (scores 0-1), *Moderate* (score 2), and *Severe* (scores 3-4). This grouping was adopted to mitigate class imbalance and to ensure sufficient samples per class for robust model training. When multiple ratings were available, we applied a majority-voting.

**Table 4:** Demographics and clinical information of patients in our dataset

Characteristics	Value
Age (Mean ± SD)	61.35 ± 8.69
Sex, Man/Woman	264 / 182
Disease duration (Mean ± SD)	31.87 ± 17.55
Medication status, On/Off	1979 / 2094
MDS-UPDRS I (Mean ± SD)	2.77 ± 2.44
MDS-UPDRS II (Mean ± SD)	8.73 ± 6.11
MDS-UPDRS III pre-med (Mean ± SD)	35.37 ± 12.80
MDS-UPDRS III post-med (Mean ± SD)	29.62 ± 12.05
MDS-UPDRS IV (Mean ± SD)	3.15 ± 3.26

Part I evaluates non-motor characteristics, including cognitive impairment, mood disturbances, sleep problems, and autonomic dysfunction. Part III consists of a motor examination conducted by a clinician, assessing rigidity, tremor, bradykinesia, and postural instability. Part IV addresses motor complications, including dyskinesias and symptom fluctuations related to medication use.

**Table 5:** Distribution of MDS-UPDRS finger-tapping scores (0-4) and the derived severity groups of the PPP dataset

MDS-UPDRS score	0	1	2	3	4
Number of patients	150	337	407	323	48
Number of videos (%)	355 (8.7 %)	1070 (26.2 %)	1380 (33.9 %)	1196 (29.4 %)	72 (1.7 %)
Merged severity groups					
Severity group	Mild (0–1)		Moderate (2)	Severe (3–4)	
Number of videos (%)	1425 (35.0 %)		1380 (33.9 %)	1268 (31.1 %)	

For classification, scores 0-1 were combined into a *Mild* category, score 2 was retained as *Moderate*, and scores 3-4 were merged into a *Severe* category.

The Personalized Parkinson Project was both conducted in accordance with the Declaration of Helsinki and Good Clinical Practice guidelines, and was approved by the local medical ethics committee (Commissie Mensgebonden Onderzoek, regio Arnhem-Nijmegen, reference number 2016-2934). All participants provided informed consent prior to enrollment.

To assess the generalizability of our method on an independent dataset, we used the TULIP^32^ dataset. This dataset comprises recordings from 11 people with PD, captured using six synchronized cameras. Each patient was recorded separately for the left and right hands. The videos were independently rated by three clinical expert neurologists, and the final scores were determined using majority voting. In this work, we used only the front-view recorded videos.

### Video preprocessing

In order to quantify distinct motor characteristics of the finger-tapping task, we first detected and cropped the participant from each video frame using an automated person detection algorithm. In this step, the videos are cropped to focus on the patient and remove redundant background information. The cropping procedure is outlined in Algorithm 1. We applied the YOLOv5^44^ person detection model to the first frame of each video to detect individuals and assign unique IDs to each person. Subsequently, a person re-identification (ReID) technique based on ResNet50 model^45^ was used to match detected individuals across frames and track their positions over time. For each detected individual, the number of frames in which they were consistently present was calculated. The individual with the longest continuous presence was identified as the patient. To define the cropping region, we selected the bounding box corresponding to the 90th percentile of the patient’s detected bounding box sizes across all frames. This approach helps to minimize the impact of potential tracking errors by excluding outlier bounding boxes. This bounding box was then enlarged by 20 % and adjusted to a square shape. Finally, all frames were cropped according to this adjusted bounding box, resulting in patient-centered video sequences.

**Algorithm 1:** Video Cropping

1: **Input:** Video file, CSV file with video names

2: **Output:** Cropped video focused on the patient

3: Load YOLOv5 model for person detection

4: Load re-identification model (ResNet50) for tracking

5: Load video names from CSV

6: **for** each video in video names **do**

7:  Initialize video capture and set output paths

8:  Process first frame to detect persons and assign IDs

9:  Extract ResNet50-based features for each detected person in first frame

10: ** while** video has frames **do**

11:   Detect persons in the current frame

12:   Extract ResNet50-based features for each detected person

13:   **for** each initially detected person **do**

14:    Match current frame’s detected persons with initial features

15: **   if** person is detected and matches criteria **then**

16:     Update last valid position and save bounding box

17: **   end if**

18: **   end for**

19:   **end while**

20:   Select the most frequently detected person ID as patient

21: Select bounding box at 90th percentile of area size

22:   Increase bounding box size by 20%

23:   Adjust bounding box to be square

24:   Center bounding box around the patient

25:   Reinitialize video capture for cropping

26:   Initialize output video with adjusted bounding box dimensions

27: **  while** video has frames **do**

28:    Crop each frame to fixed bounding box

29:    Write cropped frame to output video

30: **  end while**

31: ** end for**

### Keypoint extraction

**Fig. 8:**
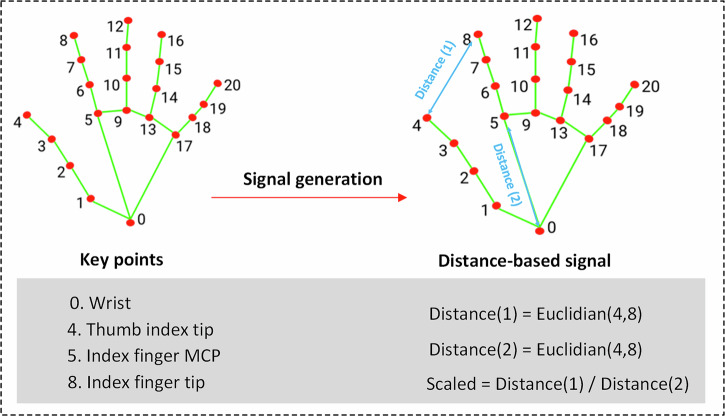
Signal generation based on the distance between the thumb and index finger. The distance-based signal is calculated as the Euclidean distance between the thumb tip and index finger tip divided by palm length.

MediaPipe^28^ was subsequently applied to the cropped frames to estimate the positions of 21 hand keypoints (Fig. 8) as it has been validated for clinical applications in multiple prior studies^46–48^. Based on these keypoints, a one-dimensional distance signal was derived by computing the Euclidean distance between the *thumb* and *index finger tips* in each frame, defined as follows:

1 $${d}_{1}({f}_{i})=\sqrt{{({{\rm{T}}}_{x}(i)-{{\rm{I}}}_{x}(i))}^{2}+{({{\rm{T}}}_{y}(i)-{{\rm{I}}}_{y}(i))}^{2}}$$

Where (*T*_*x*_(*i*), *T*_*y*_(*i*)) are the x and y coordinates of the thumb tip and (*I*_*x*_(*i*), *I*_*y*_(*i*)) the x and y coordinates of the index finger tip at frame *i*. The distance *d*_1_(*i*) is computed for every frame *i* in the video. To make the metric invariant to the distance from the camera, it is scaled by the distance between the index finger’s metacarpophalangeal (MCP) joint and the wrist keypoint as shown in Figure 8:

2 $${d}_{2}({f}_{i})=\sqrt{{({{\rm{W}}}_{x}(i)-{{\rm{MCP}}}_{x}(i))}^{2}+{({{\rm{Wrist}}}_{y}(i)-{{\rm{MCP}}}_{y}(i))}^{2}}$$

Here (*W*_*x*_(*i*), *W*_*y*_(*i*)) and (*MCP*_*x*_(*i*), *MCP*_*y*_(*i*)) describe the x and y coordinates of wrist and MCP, respectively. The scaled distance for each frame *i* is then calculated as:

3 $$s(i)=\frac{{d}_{1}(i)}{{d}_{2}(i)}$$

We applied frame-wise scaling to account for transient changes in hand orientation and distance to the camera during finger tapping, which affect both the thumb-index distance and the wrist-MCP reference distance. The distance signal over the entire video is obtained by concatenating the scaled distances across all *N* frames:

4 $$S=[s(1),s(2),\ldots ,s(N)]$$

### Quantification of PD motor characteristics

Based on the recent update in the clinical definition of bradykinesia^3^, we defined 12 interpretable features to capture the four primary PD motor deficits captured during the finger-tapping examination: hypokinesia, bradykinesia, sequence effect and hesitation-halts. In addition, we also derived two features focused on movement speed, which capture the combined effects of hypo- & bradykinesia. Below, we describe how these features are derived from the scaled signal *S*.

Hypokinesia refers to a reduced amplitude of movement (difficulty to fully open the hand). To obtain this amplitude, peaks and troughs are detected within the distance signal of equation (4) and the difference between a trough and a peak is defined as the amplitude. A peak detection algorithm^49^ is applied to the scaled distance signal to identify peaks {*p*_1_, *p*_2_, …, *p*_*M*_}, and trough values {*t*_1_, *t*_2_, …, *t*_*K*_} where *M* and *K* are the total number of detected peaks and troughs, respectively. These peaks correspond to frames when the patient fully opens their hand during tapping, whereas the troughs correspond to moments when the fingers are closed. The tapping amplitudes are then computed as follows:

5 $${{\rm{Amp}}}_{{\rm{i}}}={{\rm{p}}}_{i}-{{\rm{t}}}_{i},\,i=1,2,\ldots ,\min (M,K)$$

The hypokinesia feature is then obtained as follows:

- Average amplitude
  It indicates the average amplitude across all tapping cycles.

6 $${{\rm{Amp}}}_{{\rm{avg}}}=\frac{1}{M}\mathop{\sum }\limits_{i=1}^{M}{{\rm{Amp}}}_{i}$$

Bradykinesia refers to the slowness of voluntary movements, which results in an increased cycle duration. We defined the cycle duration as the time between consecutive peaks in the distance signal. From this, we derived the average cycle duration:

- Average cycle duration This feature measures the average time between successive peaks in the signal and it is calculated as follows:

7 $${{\rm{CD}}}_{{\rm{avg}}}=\frac{{\sum }_{j=2}^{M}\left({T}_{{p}_{j}}-{T}_{{p}_{j-1}}\right)}{M-1}$$

where ${T}_{{p}_{j}}$ is the timestamp of the *j*-th peak.

Because there is evidence that bradykinesia and hypokinesia share a common pathophysiological background, and both improve with dopaminergic medication or deep brain stimulation^3^, we added two combined brady- & hypokinesia features derived from the speed signal. Since speed is influenced by both the amplitude and cycle duration, it is a combination of hypokinesia and bradykinesia.

The speed signal is computed as the displacement between consecutive frames divided by the time between two frames and it quantifies how quickly the hands move over time:

8 $${{\rm{speed}}}_{i}=\frac{| {{\rm{s}}}_{i}-{{\rm{s}}}_{i-1}| }{\Delta t},\,\Delta t=\frac{1}{{\rm{FPS}}}$$

where *FPS* is video frame rate.

Two features are obtained in this category according to the following equations:

- Cycle Average Speed (CAS)
  The average speed for each tapping cycle is obtained as follows:

9 $${{\rm{CAS}}}_{j}=\frac{1}{({T}_{{p}_{j}}-{T}_{{p}_{j-1}})}\mathop{\sum }\limits_{t={T}_{{p}_{j-1}}+\Delta t}^{{T}_{{p}_{j}}}{\rm{Speed}}(t),\,j=1,2,\ldots ,M-1$$

The feature is then obtained through averaging across all cycles:

10 $${{\rm{CAS}}}_{{\rm{avg}}}=\frac{1}{M}\mathop{\sum }\limits_{i=1}^{M-1}{{\rm{CAS}}}_{i}$$

This feature captures the average rate at which the position of the fingers changes over time and depends on both the amplitude and the cycle duration.

- Cycle Maximum Speed (CMS)
  For each tapping cycle, 95 % speed values are obtained to account for incidental outliers that may occur due to keypoint detection:

11 $$\begin{array}{lll}{{\rm{CMS}}}_{j}={{\rm{percentile}}}_{0.95}\left(\{{\rm{Speed}}(t):{T}_{{p}_{j-1}}\le t\le {T}_{{p}_{j}}\}\right),\\\qquad\quad\,\,\,\,j=1,2,\ldots ,M-1.\end{array}$$

The metric is averaged across all cycles:

12 $${{\rm{CMS}}}_{{\rm{avg}}}=\frac{1}{M}\mathop{\sum }\limits_{i=1}^{M-1}{{\rm{CMS}}}_{i}$$

This features captures the average maximum speed achieved across tapping cycles. This feature is related to the amplitude and cycle duration, but also captures additional information because the maximum speed also depends on the shape of the tapping movement.

People with PD often struggle to maintain consistent performance during repetitive tasks such as tapping. Sequence effect is defined as a progressive reduction in amplitude and/or speed, and progressive increase in cycle duration during the task.

- Amplitude slope
  A linear trend is fitted to the sequence of amplitudes *amp*_*i*_. The slope quantifies the rate at which the amplitude declines over time.
- Cycle duration slope
  A linear trend is fitted to the sequence of cycle durations. An increasing slope reflects that the patient takes progressively more time to complete each tap.
- Speed slope
  A linear trend is fitted to the sequence of average speeds *CAS*_*j*_. The slope quantifies the rate at which average speed declines over time.

Hesitation-halts refer to increased variability in the amplitude and timing of the tapping movements. We designed five features to separately capture the coefficient of variation in amplitude, cycle duration, CAS, CMS, and the occurrence of longer interruptions:

- CV of amplitude
  This feature captures irregularities in the tapping amplitude (normalized by the average amplitude), and is calculated as:

13 $${{\rm{Amp}}}_{{\rm{CV}}}=\frac{\sqrt{\frac{{\sum }_{j=1}^{M}{\left(am{p}_{j}-{{\rm{amp}}}_{{\rm{Avg}}}\right)}^{2}}{M}}}{{{\rm{amp}}}_{{\rm{Avg}}}}$$

- CV of cycle duration
  This feature reflects irregularities in cycle duration (normalized by the average cycle duration), and is calculated as:

14 $${{\rm{CD}}}_{{\rm{CV}}}=\frac{\sqrt{\frac{{\sum }_{j=2}^{M}{\left({T}_{{p}_{j}}-{T}_{{p}_{j-1}}-{{\rm{CD}}}_{{\rm{avg}}}\right)}^{2}}{M-1}}}{{{\rm{CD}}}_{{\rm{avg}}}}$$

- CV of speed
  The following features are proposed to capture irregularities in maximum and average speed across tapping cycles:

15 $${{\rm{CMS}}}_{{\rm{CV}}}=\frac{\sqrt{\frac{{\sum }_{j=1}^{M-1}{\left(CM{S}_{j}-{{\rm{CMS}}}_{{\rm{avg}}}\right)}^{2}}{M-1}}}{{{\rm{CMS}}}_{{\rm{avg}}}}$$

16 $${{\rm{CAS}}}_{{\rm{CV}}}=\frac{\sqrt{\frac{{\sum }_{j=1}^{M-1}{\left(CA{S}_{j}-{{\rm{CAS}}}_{{\rm{avg}}}\right)}^{2}}{M-1}}}{{{\rm{CAS}}}_{{\rm{avg}}}}$$

- Number of interruptions
  Patients with PD may exhibit brief pauses, halts, or freezing during repetitive movements such as tapping^10^. To quantify them, we consider a cycle duration as an interruption if it is longer than a threshold value. The threshold value is equal to the two times the median for all cycle durations. Each interval exceeding this threshold indicates a deviation from the patient’s typical rhythmic tapping. The total number of such intervals is summed to yield the *Number of interruptions*.

### Underlying structure of motor characteristics using PCA-varimax

To investigate the underlying structure of the extracted video-based features and assess whether they align with clinically defined categories of motor deficits^3^, we applied a principal component analysis with orthogonal varimax rotation. PCA is a statistical method that transforms a set of potentially correlated variables into a set of linearly uncorrelated components ranked by the variance they capture. To enhance interpretability, we applied varimax rotation, which maximizes the variance of squared loadings within each component and ensures that each principal component is dominantly associated with a subset of features—a property that is critical for clinical interpretability. This data-driven framework allows us to evaluate whether features presumed to belong to the same clinical category (e.g., amplitude and speed decrements under the sequence effect) indeed exhibit high covariance, or whether they separate into distinct, finer-grained substructures, thereby validating or refining the clinical categorization of PD motor characteristics captured during the finger-tapping test.

### Machine learning methods for score classification

To verify the clinical validity of our approach, we evaluated whether the feature set can be used to train machine learning classifiers to estimate the MDS-UPDRS finger-tapping score. For classification, we defined three severity levels based on the original MDS-UPDRS scores: *mild* (scores 0-1), *moderate* (score 2), and *severe* (scores 3-4). We evaluated three classifiers: logistic regression^50^, LightGBM^51^, and random forest^52^. Logistic regression is a simple linear classifier that delivers (pseudo-) probabilities for each class by comparing the weighted sum of input features across all classes. The final prediction corresponds to the class with the highest probability. The model’s parameters are trained using maximum likelihood estimation (MLE) by minimizing the differences between predicted probabilities and actual labels. LightGBM constructs an ensemble of decision trees using gradient boosting, where each tree is trained to minimize the residual errors of its predecessors. The random forest classifier, in contrast, builds multiple independent decision trees by randomly sampling subsets of data points and features. The final prediction is derived from an average of all tree outputs, which typically delivers a low-variance model. We selected these classifiers to facilitate performance comparison, as logistic regression was used by Guarin et al.^29^ and LightGBM was employed in the study by Islam et al.^27^. Additionally, we included the random forest classifier as a widely used non-linear ensemble method.

In this work, both multi-class classification and ordinal classification are employed to predict MDS-UPDRS scores^53^. Multi-class classification is used to predict one of several discrete and unordered classes. Each class is treated independently, with no assumptions about relationships or order between them. In contrast, ordinal classification explicitly accounts for the ordered structure of disease severity. Following the approach of Frank et al.^53^, the ordinal problem is decomposed into *K* − 1 binary classification tasks, where *K* is the number of ordered classes. For the three severity levels considered here, two binary classifiers are trained: one separating mild from moderate and severe, and another separating severe from mild and moderate. The outputs of these classifiers are then combined to produce the final ordinal prediction.

For each classification algorithm, nested cross-validation, coupled with Optuna optimization^54^, is utilized for hyperparameter tuning. The process involves two levels of data splitting: an outer loop and an inner loop. In the outer loop, the dataset is divided into training and testing subsets using leave-one-subject-out cross-validation. Within the inner loop, the training subset is further partitioned into training and validation folds using 5-fold cross-validation stratified at the patient level. A 5-fold scheme was chosen for computational efficiency while still providing robust validation. Hyperparameter tuning is conducted within the inner folds, and the best-performing hyperparameters are applied for model evaluation in the outer loop. All classifiers are implemented and trained using the Scikit-learn library^55^.

To thoroughly evaluate the effectiveness of our proposed MDS-UPDRS classification method, we conducted a comparative analysis with several state-of-the-art approaches from recent literature. For a fair and unbiased comparison, all related works are re-trained on our dataset, and their hyperparameters are optimized. This approach ensures that any observed performance differences arise from methodological variations rather than details in data or hyperparameter tuning. The first approach for comparison is selected from Islam et al.^27^ with 116 features including speed, acceleration, amplitude, period, and frequency. A cross correlation between pair of features is applied to identify highly correlated pairs and reduce feature size. A multi-class lightGBM classifier is used. The second approach for comparison is selected from Guarin et al.^29^ with features from angular displacement signal including amplitude, speed, cycle duration, and movement rate. Multi-class, ordinal and tiered logistic regression classifications are considered for the comparison. We also compared our method with a deep learning-based approach by Lu et al.^34^ that uses raw distance and motion signals as input and trains a CNN-based model for classification.

As evaluation metrics we included accuracy, balanced accuracy, macro precision, and macro F1-score. The detailed metrics are defined as follows:

1. Accuracy: It measures the proportion of correctly predicted samples among the total number of samples:

17 $$\mathrm{Accuracy}=\frac{{\sum }_{i=1}^{K}T{P}_{i}}{{\sum }_{i=1}^{K}{P}_{i}}$$

where *TP*_*i*_ is true positives for class *i*, representing the number of samples correctly predicted as belonging to class *i*. *P*_*i*_ is total number of actual samples in class *i* and *K* is the total number of classes.

Although accuracy is widely used, it is less suitable for imbalanced datasets. To address this limitation, *balanced accuracy* is also included.

2. Balanced Accuracy: It accounts for class imbalance by averaging the accuracy for each class to provide a fair evaluation across all classes:

18 $${\rm{Balanced}}\,{\rm{ACC}}=\frac{1}{K}\mathop{\sum }\limits_{i=1}^{K}\frac{T{P}_{i}}{{P}_{i}}=\frac{1}{K}\mathop{\sum }\limits_{i=1}^{K}\frac{T{P}_{i}}{T{P}_{i}+F{N}_{i}}$$

where *FN*_*i*_ is false negatives for class *i*, representing the number of samples from class *i* incorrectly predicted as belonging to other classes.

3. Macro Precision:

Precision measures the proportion of true positive predictions among all positive predictions made by the model:

19 $${{\rm{Precision}}}_{i}=\frac{T{P}_{i}}{T{P}_{i}+F{P}_{i}}$$

where *FP*_*i*_ is false positives for class *i*, representing the number of samples incorrectly predicted as belonging to class *i*. The macro-averaged precision aggregates precision values across all classes to provide a single metric:

20 $${\mathrm{Precision}}_{\mathrm{Macro-average}}=\frac{{\sum }_{i=1}^{K}{\mathrm{Precision}}_{i}}{K}$$

This metric is crucial in scenarios where minimizing false positives is important.

4. Macro F1-Score: The F1-score is the harmonic mean of precision and recall that offers a balanced evaluation of the two metrics.

21 $$F{1}_{i}=\frac{{{\rm{Precision}}}_{i}\times {{\rm{Recall}}}_{i}}{{{\rm{Precision}}}_{i}+{{\rm{Recall}}}_{i}}$$

22 $$F{1}_{\mathrm{Macro-average}}=\frac{{\sum }_{i=1}^{K}F{1}_{i}}{K}$$

### Statistical significance testing

We performed statistical analyses at two levels:

1. Feature-level analysis to examine the relationship between video-based features and clinical ratings.
2. Classification-level analysis to compare different machine learning models.

For each extracted feature, we examined group differences across the five MDS-UPDRS finger-tapping scores (0-4). To account for potential demographic influences, a one-way analysis of covariance (ANCOVA) was performed with age and sex included as covariates. The normality of each feature was assessed using the Shapiro-Wilk test. Post-hoc pairwise comparisons were then conducted to evaluate differences between each UPDRS group with scores 1-4 and the reference group with score 0. For features that followed a normal distribution, post-hoc comparisons were performed using *t*-tests. For features that violated normality assumptions, Mann-Whitney *U* tests were used instead. For both types of tests, Bonferroni correction was applied to adjust for multiple comparisons.

To verify the clinical validity of our method and evaluate differences in classification performance, we compared the performance of our model against that of existing state-of-the-art approaches. The Wilcoxon signed-rank test, a non-parametric paired difference test, was employed to determine whether the median of the differences between paired observations is significantly different from zero. This test was applied to pairs of each subject’s individual performance scores obtained from our method and from the other methods, in order to assess whether the observed differences were statistically significant.

## Supplementary information

Supplementary Information

## Data Availability

Data from the Personalized Parkinson Project used in the present study were retrieved from the PEP database: https://pep.cs.ru.nl/index.html. The PPP data are available upon request via: ppp-data@radboudumc.nl. To protect participant privacy, all video data shared through this procedure will be face-blurred and covered by informed consent from study participants. In addition, MediaPipe-derived hand keypoint coordinates and all features extracted from these signals will be made available to facilitate reproducibility and reuse of the proposed methods. More details on the procedure can be found on the website: https://www.personalizedparkinsonproject.com/home. To ensure full compliance with privacy regulations, the illustrative images included in this paper depict the author, not patients from the study cohort. All codes used in this study were implemented in Python 3.12.3 and are available at a GitHub repository: https://github.com/AI-for-Parkinson-Lab/VideoBased-PD-Biomarkers under the Apache 2.0 license.

## References

[1] Feigin, V. L. et al. Global, regional, and national burden of neurological disorders, 1990–2016: a systematic analysis for the global burden of disease study 2016. *Lancet Neurol.* **18**, 459–480 (2019). 30879893 10.1016/S1474-4422(18)30499-X PMC6459001

[2] Morris, M. E. Movement disorders in people with parkinson disease: a model for physical therapy. *Phys. Ther.* **80**, 578–597 (2000). 10842411

[3] Bologna, M. et al. Redefining bradykinesia. *Mov. Disord.* **38**, 551 (2023). 36847357 10.1002/mds.29362 PMC10387192

[4] Bologna, M. & Guerra, A. Further insight into the role of primary motor cortex in bradykinesia pathophysiology (2023). 10.1016/j.clinph.2023.08.012 37679198

[5] Paparella, G. et al. May bradykinesia features aid in distinguishing parkinson’s disease, essential tremor, and healthy elderly individuals?. *J. Parkinson’s. Dis.* **13**, 1047–1060 (2023). 37522221 10.3233/JPD-230119 PMC10578222

[6] Bologna, M. et al. Is there evidence of bradykinesia in essential tremor?. *Eur. J. Neurol.* **27**, 1501–1509 (2020). 32396976 10.1111/ene.14312

[7] Passaretti, M. et al. The role of cerebellum and basal ganglia functional connectivity in altered voluntary movement execution in essential tremor. *Cerebellum* **23**, 2060–2081 (2024). 38761352 10.1007/s12311-024-01699-6 PMC11489212

[8] Ling, H., Massey, L. A., Lees, A. J., Brown, P. & Day, B. L. Hypokinesia without decrement distinguishes progressive supranuclear palsy from parkinson’s disease. *Brain* **135**, 1141–1153 (2012). 22396397 10.1093/brain/aws038 PMC3326257

[9] Laurencin, C. et al. Noradrenergic alterations in parkinson’s disease: a combined 11c-yohimbine pet/neuromelanin mri study. *Brain* **147**, 1377–1388 (2024). 37787503 10.1093/brain/awad338 PMC10994534

[10] Goetz, C. G. et al. Movement disorder society-sponsored revision of the unified parkinson’s disease rating scale (mds-updrs): scale presentation and clinimetric testing results. *Mov. Disord.: Off. J. Mov. Disord. Soc.* **23**, 2129–2170 (2008). 10.1002/mds.22340 19025984

[11] Richards, M., Marder, K., Cote, L. & Mayeux, R. Interrater reliability of the unified parkinson’s disease rating scale motor examination. *Mov. Disord.* **9**, 89–91 (1994). 8139610 10.1002/mds.870090114

[12] Berlot, R., Rothwell, J. C., Bhatia, K. P. & Kojović, M. Variability of movement disorders: the influence of sensation, action, cognition, and emotions. *Mov. Disord.* **36**, 581–593 (2021). 33332680 10.1002/mds.28415

[13] Angelini, L., Paparella, G. & Bologna, M. Distinguishing essential tremor from parkinson’s disease: clinical and experimental tools. *Expert Rev. Neurotherapeutics* **24**, 799–814 (2024). 10.1080/14737175.2024.2372339 39016323

[14] Guerra, A., D’Onofrio, V., Ferreri, F., Bologna, M. & Antonini, A. Objective measurement versus clinician-based assessment for parkinson’s disease. *Expert Rev. Neurotherapeutics* **23**, 689–702 (2023). 10.1080/14737175.2023.2229954 37366316

[15] Huckvale, K., Venkatesh, S. & Christensen, H. Toward clinical digital phenotyping: a timely opportunity to consider purpose, quality, and safety. *NPJ digital Med.* **2**, 1–11 (2019). 10.1038/s41746-019-0166-1 PMC6731256 31508498

[16] Tarolli, C. G. et al. Feasibility, reliability, and value of remote video-based trial visits in parkinson’s disease. *J. Parkinson’s. Dis.* **10**, 1779–1786 (2020). 32894251 10.3233/JPD-202163

[17] Larson, D. N., Schneider, R. B. & Simuni, T. A new era: the growth of video-based visits for remote management of persons with parkinson’s disease. *J. Parkinson’s. Dis.* **11**, S27–S34 (2021). 33492246 10.3233/JPD-202381 PMC8385503

[18] Li, H., Shao, X., Zhang, C. & Qian, X. Automated assessment of parkinsonian finger-tapping tests through a vision-based fine-grained classification model. *Neurocomputing* **441**, 260–271 (2021).

[19] Williams, S. et al. Supervised classification of bradykinesia in parkinson’s disease from smartphone videos. *Artif. Intell. Med.* **110**, 101966 (2020). 33250146 10.1016/j.artmed.2020.101966

[20] Deng, D. et al. Interpretable video-based tracking and quantification of parkinsonism clinical motor states. *npj Parkinson’s. Dis.* **10**, 122 (2024). 38918385 10.1038/s41531-024-00742-x PMC11199701

[21] Yu, T., Park, K. W., McKeown, M. J. & Wang, Z. J. Clinically informed automated assessment of finger tapping videos in parkinson’s disease. *Sensors* **23**, 9149 (2023). 38005535 10.3390/s23229149 PMC10674854

[22] Khan, T., Nyholm, D., Westin, J. & Dougherty, M. A computer vision framework for finger-tapping evaluation in parkinson’s disease. *Artif. Intell. Med.* **60**, 27–40 (2014). 24332155 10.1016/j.artmed.2013.11.004

[23] Skaramagkas, V., Pentari, A., Kefalopoulou, Z. & Tsiknakis, M. Multi-modal deep learning diagnosis of parkinson’s disease—a systematic review. *IEEE Trans. Neural Syst. Rehabilitation Eng.* **31**, 2399–2423 (2023). 10.1109/TNSRE.2023.3277749 37200116

[24] Amo-Salas, J. et al. Computer vision for parkinson’s disease evaluation: A survey on finger tapping. In *Healthcare* 12, 439 (MDPI, 2024). 10.3390/healthcare12040439 PMC10888014 38391815

[25] Zhao, Z. et al. Time series clustering to examine presence of decrement in parkinson’s finger-tapping bradykinesia. In *2020 42nd Annual International Conference of the IEEE Engineering in Medicine & Biology Society (EMBC)*, 780–783 (IEEE, 2020). 10.1109/EMBC44109.2020.9175638 33018102

[26] Heye, K. et al. Validation of computer vision technology for analyzing bradykinesia in outpatient clinic videos of people with parkinson’s disease. *J. Neurological Sci.* **466**, 123271 (2024). 10.1016/j.jns.2024.123271 39476714

[27] Islam, M. S. et al. Using ai to measure parkinson’s disease severity at home. *npj Digital Med.* **6**, 156 (2023). 10.1038/s41746-023-00905-9 PMC10444879 37608206

[28] Lugaresi, C. et al. Mediapipe: A framework for building perception pipelines (2019).

[29] Guarin, D. L., Wong, J. K., McFarland, N. R. & Ramirez-Zamora, A. Characterizing disease progression in parkinson’s disease from videos of the finger tapping test. *IEEE Transactions on Neural Systems and Rehabilitation Engineering* (2024). 10.1109/TNSRE.2024.3416446 PMC11260436 38905096

[30] Guo, Z. et al. Vision-based finger tapping test in patients with parkinson’s disease via spatial-temporal 3d hand pose estimation. *IEEE J. Biomed. Health Inform.* **26**, 3848–3859 (2022). 35349459 10.1109/JBHI.2022.3162386

[31] Redmon, J. Yolov3: An incremental improvement. *arXiv preprint arXiv:1804.02767* (2018).

[32] Kim, K., Lyu, S., Mantri, S. & Dunn, T. W. Tulip: Multi-camera 3d precision assessment of parkinson’s disease. In *Proceedings of the IEEE/CVF Conference on Computer Vision and Pattern Recognition*, 22551–22562 (2024).

[33] Amprimo, G., Masi, G., Olmo, G. & Ferraris, C. Enhancing model generalizability in parkinson’s disease automatic assessment: A semi-supervised approach across independent experiments. In *2024 46th Annual International Conference of the IEEE Engineering in Medicine and Biology Society (EMBC)*, 1–4 (IEEE, 2024). 10.1109/EMBC53108.2024.10781915 40039364

[34] Lu, M. et al. Quantifying parkinson’s disease motor severity under uncertainty using mds-updrs videos. *Med. image Anal.* **73**, 102179 (2021). 34340101 10.1016/j.media.2021.102179 PMC8453121

[35] Yang, Y.-Y. et al. Fasteval parkinsonism: an instant deep learning–assisted video-based online system for parkinsonian motor symptom evaluation. *npj Digital Med.* **7**, 31 (2024). 10.1038/s41746-024-01022-x PMC10853559 38332372

[36] Yang, N. et al. Automatic detection pipeline for accessing the motor severity of parkinson’s disease in finger tapping and postural stability. *IEEE Access* **10**, 66961–66973 (2022).

[37] Kaiser, H. F. The varimax criterion for analytic rotation in factor analysis. *Psychometrika* **23**, 187–200 (1958).

[38] Guarín, D. L. et al. What the trained eye cannot see: Quantitative kinematics and machine learning detect movement deficits in early-stage parkinson’s disease from videos. *Parkinsonism Relat. Disord.* **127**, 107104 (2024). 39153421 10.1016/j.parkreldis.2024.107104 PMC13501229

[39] Kim, J.-W. et al. Quantification of bradykinesia during clinical finger taps using a gyrosensor in patients with parkinson’s disease. *Med. Biol. Eng. Comput.* **49**, 365–371 (2011). 21052856 10.1007/s11517-010-0697-8

[40] Okuno, R., Yokoe, M., Akazawa, K., Abe, K. & Sakoda, S. Finger taps movement acceleration measurement system for quantitative diagnosis of parkinson’s disease. In *2006 International Conference of the IEEE Engineering in Medicine and Biology Society*, 6623–6626 (IEEE, 2006). 10.1109/IEMBS.2006.260904 17959469

[41] Guarín, D. L. et al. Video analysis reveals early signs of bradykinesia in rem sleep behavior disorder and parkinson’s disease. *npj Parkinson’s. Dis.* **11**, 222 (2025). 40739299 10.1038/s41531-025-01082-0 PMC12311115

[42] Hey, G. E. et al. Developing personalized treatment strategies for parkinson’s disease based on disease subtypes. *Expert Review of Neurotherapeutics* (2025). 10.1080/14737175.2025.2552784 40888598

[43] Bloem, B. et al. The personalized parkinson project: examining disease progression through broad biomarkers in early parkinson’s disease. *BMC Neurol.* **19**, 1–10 (2019). 31315608 10.1186/s12883-019-1394-3 PMC6636112

[44] Jocher, G. YOLOv5 by ultralytics (2020).

[45] Zhou, K., Yang, Y., Cavallaro, A. & Xiang, T. Omni-scale feature learning for person re-identification. In *Proceedings of the IEEE/CVF international conference on computer vision*, 3702–3712 (2019).

[46] Amprimo, G. et al. Hand tracking for clinical applications: Validation of the google mediapipe hand (gmh) and the depth-enhanced gmh-d frameworks. *Biomed. Signal Process. Control* **96**, 106508 (2024).

[47] Maggioni, V., Azevedo-Coste, C., Durand, S. & Bailly, F. Optimisation and comparison of markerless and marker-based motion capture methods for hand and finger movement analysis. *Sensors* **25**, 1079 (2025). 40006308 10.3390/s25041079 PMC11858933

[48] Shenoy, P., Sundaram, S. M., Manikandan, N. et al. Multi-plane comparative study of finger angles and range of motion using mediapipe pose estimation for kinematic analysis. In *2025 International Conference on Biomedical Engineering and Sustainable Healthcare (ICBMESH)*, 1–6 (IEEE, 2025).

[49] Virtanen, P. et al. Scipy 1.0: fundamental algorithms for scientific computing in python. *Nat. methods* **17**, 261–272 (2020). 32015543 10.1038/s41592-019-0686-2 PMC7056644

[50] Menard, S.*Applied logistic regression analysis* (SAGE publications, 2001).

[51] Ke, G. et al. Lightgbm: A highly efficient gradient boosting decision tree. *Advances in neural information processing systems* **30** (2017).

[52] Breiman, L. Random forests. *Mach. Learn.* **45**, 5–32 (2001).

[53] Frank, E. & Hall, M. A simple approach to ordinal classification. In *Machine Learning: ECML 2001: 12th European Conference on Machine Learning Freiburg, Germany, September 5–7, 2001 Proceedings 12*, 145–156 (Springer, 2001).

[54] Akiba, T. et al. Optuna: A next-generation hyperparameter optimization framework. In *Proceedings of the 25th ACM SIGKDD International Conference on Knowledge Discovery & Data Mining*, 2623–2631 (2019).

[55] Pedregosa, F. et al. Scikit-learn: Machine learning in python. *J. Mach. Learn. Res.* **12**, 2825–2830 (2011).

