# Supplementary material for: Interpretable and granular video-based quantification of motor characteristics from the finger-tapping test in Parkinson’s disease
Source: NPJ Parkinsons Dis. 2026 Mar 8;12:101. doi: 10.1038/s41531-026-01307-w (PMC13100129; doi:10.1038/s41531-026-01307-w)
Supplement: Supplementary file 1 — Supplementary Information [file 41531_2026_1307_MOESM1_ESM.pdf]

Supplementary Material

Supplementary Table

Supplementary Table 1: **Train and test on Personalized Parkinson Project (PPP) dataset** . The column labeled *All* reflects the combined evaluation of OFF and ON medication states. Across all classifiers and evaluation metrics, performance in the ON medication state was consistently slightly higher than in the OFF state. This difference is likely related to the reduced motor impairment in the ON condition, which may facilitate more accurate hand pose estimation.

	Accuracy			Balanced Acc			Macro Precision			Macro F1		
	All	Off	On	All	Off	On	All	Off	On	All	Off	On
**Multi-class classification**												
Logistic regression	56.39	**55.95**	58.13	56.19	**54.63**	57.96	56.16	54.94	57.53	55.75	54.56	57.32
LightGBM	56.44	55.95	56.57	56.33	54.81	56.57	55.83	54.50	55.87	55.82	54.54	55.95
Random forest	55.16	54.35	56.71	55.13	53.36	56.72	54.79	53.29	56.12	54.84	53.30	56.29
**Ordinal classification**												
Logistic regression	56.10	55.79	57.85	55.83	54.63	57.58	56.52	**55.65**	57.70	55.74	**54.88**	57.28
LightGBM	**57.19**	55.54	**58.84**	**57.16**	54.73	**58.79**	**57.00**	54.57	**58.80**	**56.98**	54.64	**58.69**
Random forest	55.21	54.19	57.14	55.19	53.05	57.17	54.39	52.52	56.37	54.55	52.71	56.59
**Baselines**												
Random guess		33.32			33.32			33.32			33.30	
Majority class		34.84			33.33			11.61			17.23	

Supplementary Figures and Supplementary Results

Stability of the PCA-derived structure

DBSCAN is used as an auxiliary method to assess the stability of the PCA-derived structure across bootstrap resamples (by applying it to the PCA loading matrices). Bootstrapping was performed at the subject level (sampling participants with replacement and retaining all of their videos) to preserve within-subject dependencies and to test the robustness of the results against changes in dataset. For each of 1,000 bootstrap resamples, PCA was applied to the feature matrix $\mathbf{X}^{(b)} \in \mathbb{R}^{n_b \times 12}$, where n_b is the number of videos in the resample and 12 corresponds to the extracted features. This yielded a loading matrix $\mathbf{P}^{(b)} \in \mathbb{R}^{12 \times 12}$. Based on elbow criteria, 6 components are retained and subsequently rotated using orthogonal varimax rotation to enhance interpretability. Across bootstraps, all rotated loading matrices were aggregated, resulting in a collection of 6,000 loading components (6 components × 1,000 bootstraps). These vectors were clustered using the density-based DBSCAN algorithm, with absolute cosine distance as similarity measure. This approach groups features that consistently co-load on the same components across bootstrap samples, while leaving unstable assignments labeled as noise. The resulting clusters therefore represent stable latent dimensions of motor behavior. The epsilon parameter was set to correspond to an angular deviation of 30 degrees, and the minimum number of samples was set to 30. To assess the robustness of these choices, we performed a sensitivity analysis in which both parameters were varied between 10 and 45. Across this entire range of values, the resulting number of clusters remained stable, consistently yielding six dominant clusters. Notably, no components were labeled as noise, indicating that the loading patterns were highly consistent across bootstrap samples and that no additional patterns emerged in individual resamples. Supplementary Fig. 2 B projects the bootstrapped loading vectors onto a unit sphere, where data are normalized to unit length. This representation highlights the angular separation between clusters and illustrates how consistently loadings converge to distinct directions across resamples.

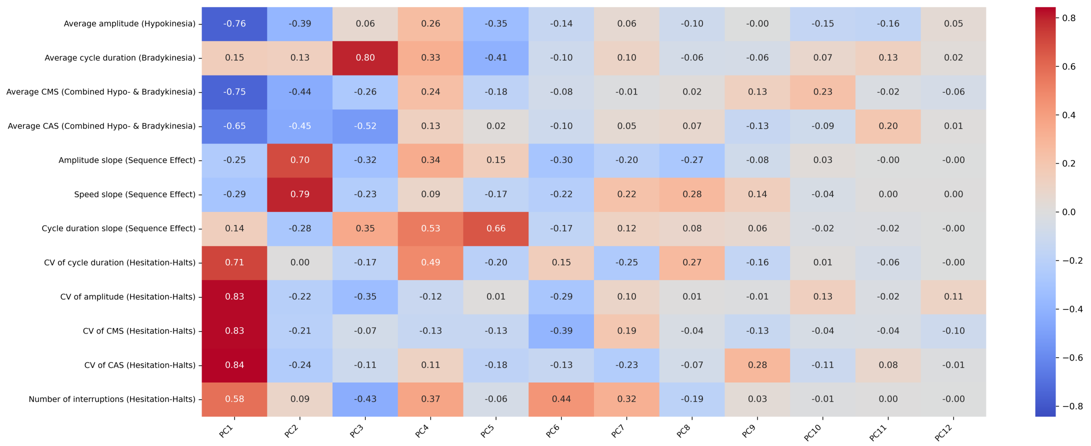

Supplementary Fig. 1: **PCA loadings for the 12 finger-tapping features.** The heatmap visualizes the loading strength of each feature.

s

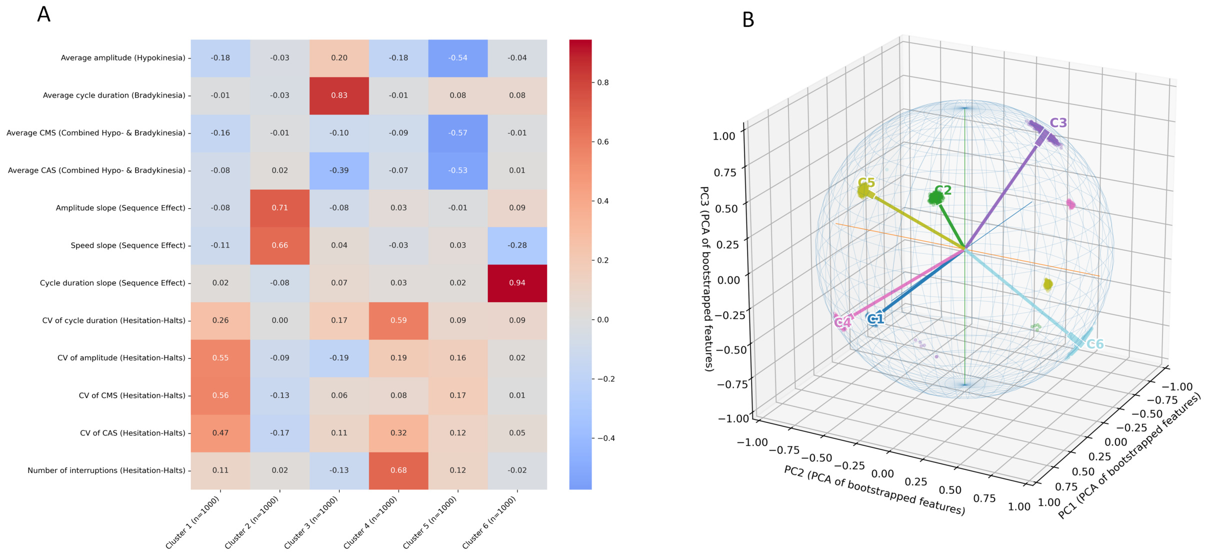

Supplementary Fig. 2: **Bootstrap principal component analysis (PCA) with varimax rotation and density-based spatial clustering of applications with noise (DBSCAN) of feature loadings. A** The heatmap displays the centroid loadings of 6 stable clusters derived from 1,000 bootstrap resamples. Warmer colors (red) indicate strong positive loadings, whereas cooler colors (blue) represent strong negative loadings. Each cluster reflects a group of features that consistently co-vary across resamples. **B** The unit-sphere projection of bootstrapped loading vectors provides a geometric view of the clustering results. Here, loadings are normalized to unit length and plotted on the sphere, where angular separation between vectors highlights distinct clusters and their stability across resamples.

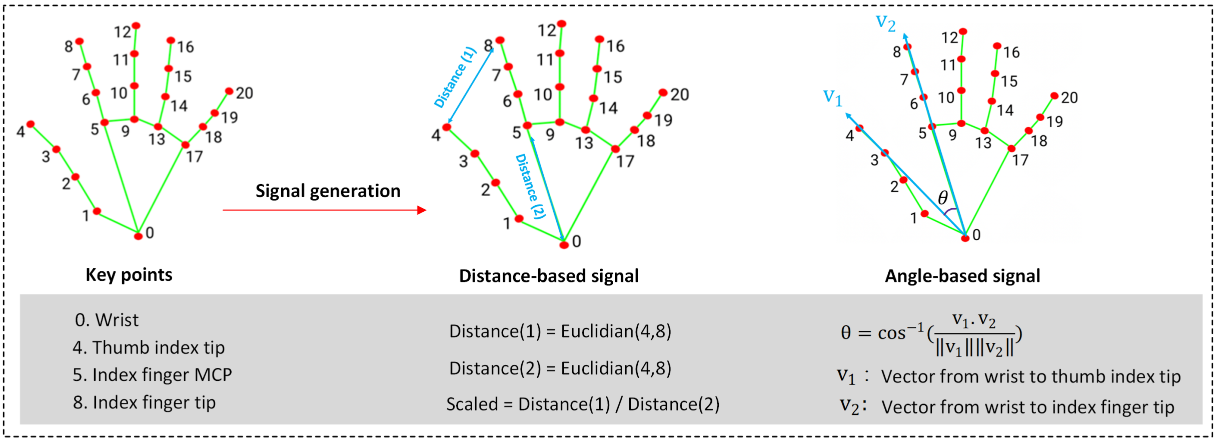

Supplementary Fig. 3: **Signal generation based on the distance between the thumb and index finger (middle) and the angle between the thumb and index finger (right)**. Distance-based representations quantify the distance between the thumb tip and index finger tip. It is calculated as the Euclidean distance between the thumb tip and index finger tip divided by palm length. In contrast, angle-based representations characterize movement through the orientation of vectors formed between the wrist and finger landmarks. The angle-based signal computed as the angle formed between vectors from the wrist to the thumb and wrist to the index finger. While both representations are derived from the same hand keypoints, they encode fundamentally different geometric properties of the movement, which can lead to different sensitivities to camera viewpoint and hand orientation.

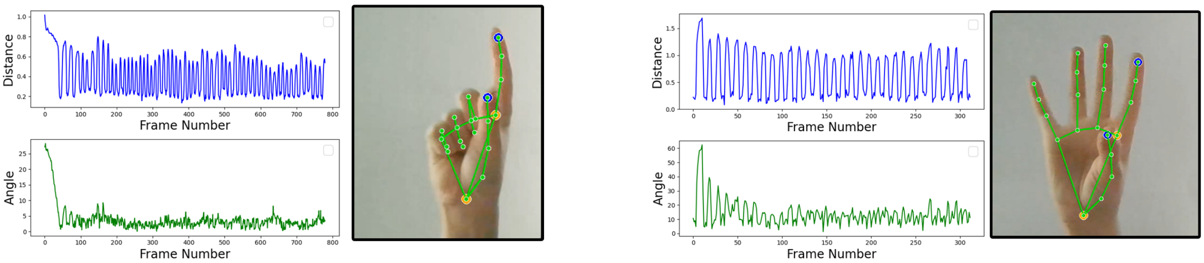

Supplementary Fig. 4: **Examples of a camera viewpoint effect where angle-based signals (green) are less accurate compared to distance-based signals (blue)**. The wrist, thumb, and index finger are nearly aligned in a straight line during the tapping task. Consequently, the vectors used in angle computation are overlapping, resulting in an angle close to zero throughout the sequence. This leads to a flat or minimally varying angular signal that fails to reflect the dynamics of finger-tapping. In contrast, the distance-based signal remains sensitive to variations in hand opening and closing.

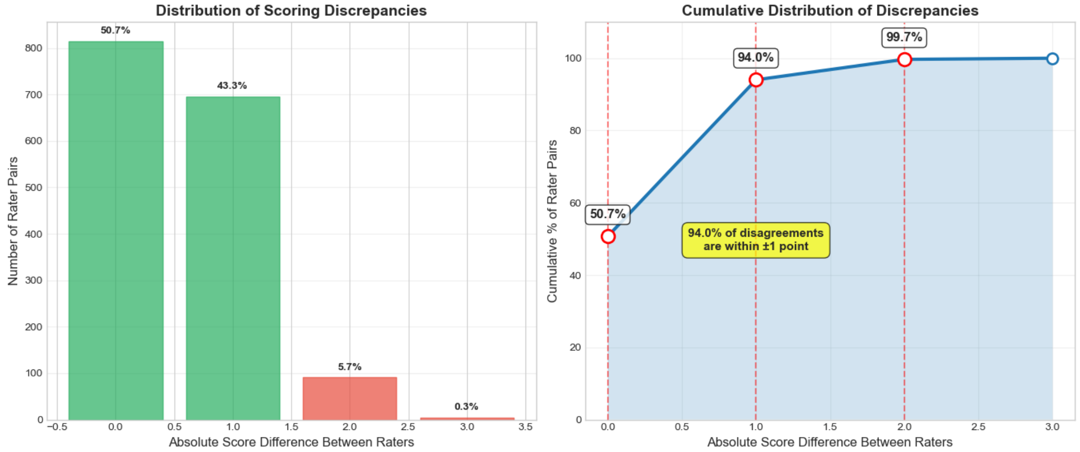

Supplementary Fig. 5: **Distribution of scoring discrepancies between raters.**(Left) Histogram showing absolute score differences for all 1,607 rater pairs across 435 videos. The y-axis indicates the number of rater pairs exhibiting each level of disagreement. Perfect agreement (difference = 0) occurred in 50.7% of pairs, while 43.3% differed by exactly ±1 point. Only 6.0% differing by 2 or more points. (Right) Cumulative distribution demonstrating that 94% of rater pairs agreed within ±1 point.

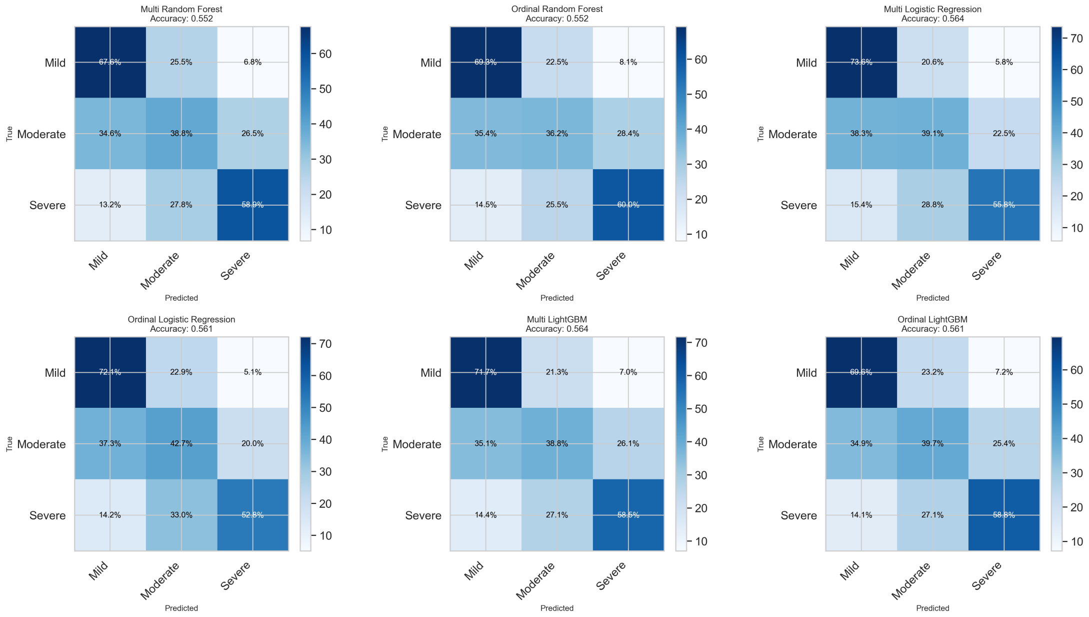

Supplementary Fig. 6: Confusion matrix for all the combinations of classifiers and classification type for our proposed method based on distance-based signal.

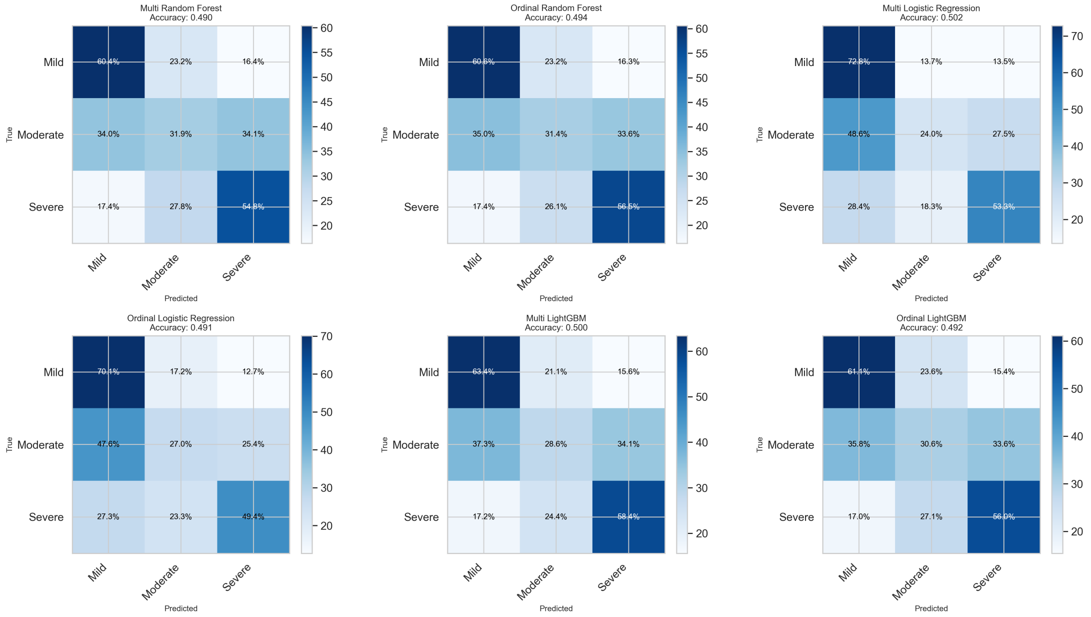

Supplementary Fig. 7: Confusion matrix for all the combinations of classifiers and classification type for angle-based signal.

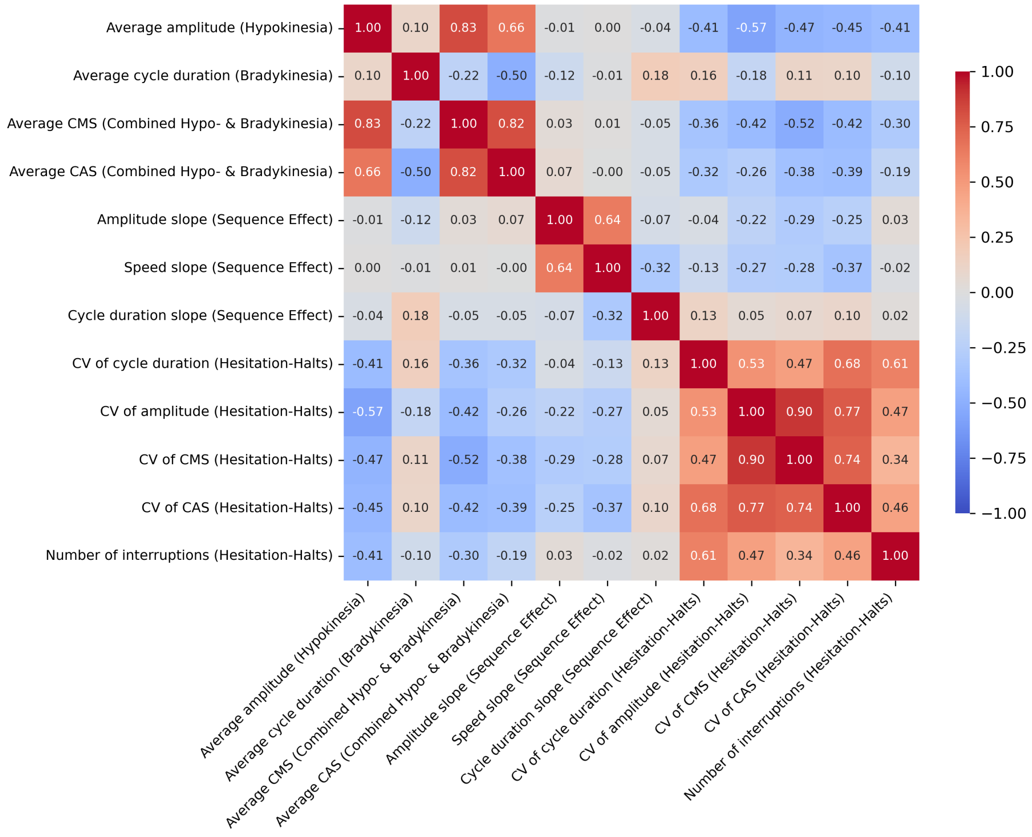

Supplementary Fig. 8: **Pearson correlation between all pairs of finger-tapping features**
